# Association of a Healthy Lifestyle with All-Cause, Cause-Specific Mortality and Incident Cancer among Individuals with Metabolic Syndrome: A Prospective Cohort Study in UK Biobank

**DOI:** 10.3390/ijerph19169936

**Published:** 2022-08-11

**Authors:** E Wu, Jun-Tao Ni, Zhao-Hui Zhu, Hong-Quan Xu, Lin Tao, Tian Xie

**Affiliations:** 1 School of Pharmacy, Hangzhou Normal University, Hangzhou 311121, China; 2 Key Laboratory of Elemene Class Anti-Cancer Chinese Medicines, Engineering Laboratory of Development and Application of Traditional Chinese Medicines, Collaborative Innovation Center of Traditional Chinese Medicines of Zhejiang Province, Hangzhou Normal University, Hangzhou 311121, China; 3 Women’s Hospital School of Medicine Zhejiang University, Hangzhou 310006, China

**Keywords:** metabolic syndrome (MetS), UK biobank, cancer incidence, healthy lifestyle, all-cause mortality, cause-specific mortality

## Abstract

This study investigated the association between a healthy lifestyle with all-cause, cause-specific mortality, and cancer incidence among individuals with metabolic syndrome (MetS). Healthy lifestyle scores were created based on MetS management guidelines, including never/quitting smoking, moderate drinking, good sleep, healthy diet, sufficient exercise, social support, and less sedentary behaviour. Weighted healthy lifestyle scores were further constructed and classified into three groups: unfavourable (lowest quintile), intermediate (quintiles 2–4), and favourable (highest quintile) lifestyles. We included 87,342 MetS participants from the UK Biobank. Hazard ratios (HRs) and 95% confidence intervals (CIs) were calculated using multivariate-adjusted Cox proportional hazards regression. During a median follow-up of 12.54 years, 6739 deaths were reported; during a median follow-up of 10.69 years, 10,802 new cancer cases were documented. We found a favourable lifestyle was inversely associated with all-cause mortality (HR: 0.57; 95%CI: 0.53–0.62), cause-specific mortality from respiratory disease, cancer, digestive disease, cardiovascular disease (HR < 1; *p*-trend < 0.001), and overall cancer incidence (HR: 0.84; 95% CI: 0.79–0.90). Our results indicate that adherence to healthy lifestyles is associated with lower overall cancer incidence and all-cause mortality risk among MetS individuals. However, causality cannot be made due to the nature of observational studies.

## 1. Introduction

Metabolic syndrome (MetS) is a public health problem as well as a clinical problem, with nearly a quarter of the world’s population suffering from it [1,2]. It is characterised by a cluster of metabolic disorders, including elevated blood pressure (BP), elevated fasting glucose, dyslipidemia, and abdominal obesity [3]. The hazards of MetS lie in its associated increased medical costs [4], increased risk of all-cause mortality [5], cardiovascular disease (CVD), cancer, and type 2 diabetes mellitus (T2DM), among others [6,7]. Therefore, interest is increasing in the prevention of adverse outcomes and premature death in individuals with MetS by targeting modifiable factors.

The international panel’s therapeutic guidelines for MetS advocates for lifestyle management [8,9], which has proven efficacy in the prevention and control of CVD, T2DM, and hypertension [10,11,12]. However, previous studies have mainly targeted limited lifestyle management, such as smoking, alcohol consumption, and exercise, and rarely considered emerging factors such as diet, sleep, social support, and sedentary behaviour [13,14]. Additionally, most studies on lifestyle management involving individuals with MetS have focused on CVD-related diseases [15] or site-specific cancers, such as endometrial cancer [16]. Research on all-cause and cause-specific mortality and the overall incidence of cancer are limited. Given the high mortality and cancer risk in individuals with MetS [5,17], it is critical to investigate the potential of modifiable lifestyles to prevent death and cancer incidence in individuals with MetS.

In this study, we used data from the UK Biobank to investigate associations between a healthy lifestyle (including never or quitting smoking, moderate drinking, good sleep, healthy diet, sufficient exercise, social support, and less sedentary behaviour) and all-cause mortality, mortality from cancer, CVD, respiratory and digestive disease, as well as incident cancer among individuals with MetS.

## 2. Materials and Methods

### 2.1. Data Source and Study Participants

This study obtained data from the UK Biobank. The UK Biobank, an ongoing cohort, has recruited more than 0.5 million individuals between 37 and 70 years across the UK. Participants’ demographic characteristics, biological samples, and potential health-related information were investigated between 2006 and 2010, and their health and medical information were continually followed up [18]. The inclusion criteria for this study were: (1) individuals with MetS at recruitment and the baseline age ≥40 years. The exclusion criteria were: (1) individuals diagnosed with cancer at baseline, (2) who died or had cancer within the first three years after recruitment, and (3) lost to follow-up or incomplete baseline information. Among about 0.5 million participants, 137,591 participants with MetS were included. We further excluded those with cancers at baseline (*n* = 12,317), lost to follow-up (*n* = 296), and those aged <40 years (*n* = 2). In addition, a total of 30,041 participants with missing information and 7593 participants who died or had cancer within the first three years after recruitment were also excluded. Overall, 87,342 participants with MetS at baseline were included in this study (Supplementary Material Figure S1).

The UK Biobank was approved by the Research Ethics Committees of the Northwest Multi-Centre (reference no. 16/NW/0274). All the study participants signed an informed consent form. This study was performed under the approval of the UK Biobank (Application number: 78563).

### 2.2. Ascertaining MetS

MetS was defined as the occurrence of three or more of the following risk factors according to the most recent joint interim statement of the International Diabetes Federation (IDF) and National Heart, Lung and Blood Institute (NHLBI)/American Heart Association (AHA): central obesity, elevated BP, increased fasting blood glucose (FBG), reduced high-density lipoprotein cholesterol (HDL-C), and increased triglycerides (TG) [19]. Waist circumference thresholds, which determined central obesity, were measured according to sex and population specifications, such as ≥85 cm for men and ≥80 cm for women in China, and ≥102 cm for men and ≥88 cm for women in Europe [19]. An elevated FBG level was defined as FBG ≥ 100 mg/dL, or a diagnosis of diabetes mellitus (DM), or the use of DM medication. Elevated BP was defined as systolic BP ≥ 130 mmHg and/or a diastolic BP ≥ 85 mmHg, a diagnosis of hypertension, or a history of antihypertensive drug treatment. Lower HDL-C level was defined as HDL-C < 1.0 mmol/L for men and < 1.3 mmol/L for women or with a history of medical treatment for reduced HDL-C. Raised TG was defined as TG ≥ 1.7 mmol/L or treatment history.

### 2.3. Assessment of Healthy Lifestyles

Healthy lifestyle scores were created based on seven recommended lifestyle behaviours (moderate alcohol consumption, never/quit smoking, good sleep, healthy diet, sufficient physical activity, social support, and less sedentary behaviour) [9,20], obtained at recruitment through touchscreen questionnaires. Moderate alcohol consumption was defined as alcohol consumption of ≤14 g/day and ≤28 g/day for women and men, respectively [21]. Never smoked and quitting smoking were classified as healthy levels [22]. Good sleep was defined as a sleep duration of 7–8 h/night without snoring [23]. A healthy diet was defined as containing at least four of the recommended food intakes as follows: adequate intake of fruits, vegetables, fish, and whole grains; lower intake of processed meats, unprocessed red meats, and refined grains [24]. Sufficient exercise was defined as moderate exercise ≥150 min/week or ≥5 d/week, vigorous exercise ≥75 min/week or at least once a week, or an equivalent combination [25,26]. Social support was described as having at least two of the following social connections: friend/family visits at least once a week, being able to confide in someone close, having a close relationship with family, or having leisure/social activities [27,28]. Less sedentary behaviour was defined as the sum of time spent driving, watching TV, and using a computer ≤4 h/day [29,30]. Participants were given one point for each of the recommended behaviours. The total score possible ranged from 0 to 7 points, with a higher score indicating a healthier lifestyle (Supplementary Material Table S1).

The weighted healthy lifestyle scores were constructed by using the original binary lifestyle factors as below: weighted healthy lifestyle scores = (β_−1_ × factor_−1_ + β_−2_ × factor_−2_ + …+ β_−7_ × factor_−7_) ÷ (β_−1_ + β_−2_ +…+ β_−7_) × 7 [24]. The βcoefficients represented the association of each binary lifestyle factor with the outcome of all-cause mortality or overall cancer incidence that were assessed by using Cox regression with adjustment for model three as below. The weighted healthy lifestyle scores were subsequently categorised as: unfavourable (lowest quintile), intermediate (quintiles 2–4), and favourable (highest quintile) lifestyles.

### 2.4. Ascertaining the Outcomes

The main outcome of this study was all-cause mortality. Mortality information was recorded by the death registry, which is linked to the National Health Service (NHS) Digital Centre (participants from England/Wales) and NHS Central Register (participants from Scotland) [31]. The cause of death was based on the 10th revision of the International Classification of Diseases (ICD-10), which included cancer (C00-C97), CVD (I05-I89), respiratory disease (J09-J99), digestive disease (K20-K93), and others. The follow-up time on the outcome of mortality, which was measured in person-years, was calculated from baseline to the date of death, or until 30 September 2021 (England/Wales participants) or 31 October 2021 (Scotland participants).

The secondary outcome was the first occurrence of incident cancer. The details of cancer incidence were obtained from the cancer registry, which is linked to the Medical Research Information Service of the NHS (participants from England/Wales) and the Information Services Division of the NHS Scotland (participants from Scotland). Death and cancer registries of UK Biobank have been proven to have high integrity and reliability [32,33]. Incident cancers were recorded using the ICD-10 (C00–C97), ICD-9 (140–208), or self-reported cancer (−1 to 9999). The follow-up of the outcome of incident cancer, as measured in person-years, was calculated from baseline to the first occurrence of cancer, death for any reason, or until 31 October 2015 (participants in Scotland) or 29 February 2020 (England/Wales participants), whichever occurred first (Supplementary Material Table S2).

### 2.5. Covariates

Covariates were collected through touchscreen questionnaires at recruitment. These included sociodemographic information, such as age (years), sex (male or female), ethnicity (white, Asian, black, or mixed), education (college/university degree, others), employment status (employed, unemployed), Townsend deprivation index (TDI) (lowest quintile, quintiles 2–4, highest quintile); health status, such as BMI, medication treatment history for anti-cholesterol drug (yes, no), antihypertensive drug (yes, no), insulin (yes, no), family history of hypertension (yes, no), DM (yes, no), or cancer (yes, no); and the number of MetS component traits (3, 4, or 5). TDI is a measure of socioeconomic status that combines information on unemployment, non-car ownership, non-homeownership, and household overcrowding at baseline, with a higher score indicating a lower socioeconomic status [34]. BMI, an international standard for measuring body fat, was calculated using the formula: BMI = weight (kg)/height (m)^2^, and divided into three groups: <25 kg/m^2^ (underweight or normal weight), 25–30 kg/m^2^ (overweight), and 30 kg/m^2^ (obese), according to the World Health Organization [35]. The participants’ weight and height were measured by well-trained nurses.

### 2.6. Statistical Analysis

The baseline characteristics of the participants were summarised based on all-cause mortality and cancer incidence. Continuous variables were presented as mean ± standard deviation (normally distributed) or median (interquartile range) (non-normally distributed) and were examined using Student’s *t*-test or Wilcoxon rank test. Categorical variables were presented as frequencies (%) and analysed using Pearson’s χ^2^ test. It should be mentioned that even minor differences can be statistically significant in a large sample size. The median follow-up person-years were calculated from the baseline survey to the date of each endpoint using the reverse Kaplan–Meier method.

Cox proportional hazard regressions were conducted to examine correlations between lifestyle factors and categories, all-cause mortality, cause-specific mortality, and incident cancer. The results were presented as hazard ratios (HRs) and 95% confidence intervals (CIs). We included age at baseline (years), sex (male, female), and ethnicity (white, Asian, black, or mixed) in model one and additionally adjusted for education (college/university degree, others), employment status (employed, unemployed), and TDI (lowest quintile, quintiles 2–4, highest quintile) in model two, and further adjusted for BMI category, medication treatment history for anti-cholesterol drug (yes, no), antihypertensive drug (yes, no), and insulin (yes, no), family history of hypertension (yes, no), DM (yes, no), cancer (yes, no), and the number of MetS components (3, 4, and 5) in model three. We performed the Schoenfeld residuals test to examine the proportional hazards assumption, and no evidence of disproportionality was found (Supplementary Material Figures S2–S7) [36]. Stratified and crossover analyses were performed according to age at baseline; sex; education; employment status; TDI; BMI; medication use history of anti-cholesterol drugs, antihypertensive drugs, and insulin; and family history of hypertension, DM, and cancer. Interactions were examined using the interaction term beta in the entire model. The population-attributable risk percent (PAR%) was calculated using the following formula [37]:

$$\mathrm{PAR}\%=\frac{P_{e}(\mathrm{HR}-1)}{P_{e}(\mathrm{HR}-1)+1}\times 100\%$$

where *P_e_* is the proportion of the population exposed to the non-low-risk group, and HR is the multivariate-adjusted Cox regression hazard ratio of those participants in the full models.

Sensitivity analyses were performed to examine the robustness of the estimates. First, given the large sample size, we excluded participants with missing values of subjective data to avoid imputations affecting the authenticity of the subjective data. Second, we excluded participants with baseline cancer, those who died, or those who were diagnosed with cancer in the first three years of follow-up to rule out potential reverse causation. Third, the unweighted healthy lifestyle scores were classified into three groups: scoring 0–2, 3–5, and 6–7, to investigate all-cause mortality, cause-specific mortality, and incident cancer risk. Fourth, we reconstructed lifestyle scores by defining 18.5 ≤ BMI < 25 kg/m^2^ as the recommended level. Fifth, never smoking was redefined as recommended behaviour. All statistical analyses were performed using Stata (version 15.0; Stata Corp, College Station, TX, USA), and graphs were plotted using R (version 4.1.3; R Core Team (2022). R Foundation for Statistical Computing, Vienna, Austria). All *p*-values were two-sided, and *p*-values < 0.05 were considered statistically significant.

## 3. Results

### 3.1. Baseline Characteristics

The baseline characteristics of participants, according to all-cause mortality and overall cancer incidence, are presented in Table 1. Among the 87,342 participants with MetS, 6739 deaths with a median follow-up period of 12.54 years (1,074,630 total person-years), and 10,802 new cases of cancer with a median follow-up period of 10.69 years (882,973 total person-years) were documented. Compared to surviving participants, those who died were more likely to be older, male, unemployed, have lower educational levels, higher BMI, and lower socioeconomic status (*p* < 0.05). The characteristics of participants with MetS excluded from the analysis are shown in Supplementary Material Table S3. Lifestyle scores were normally distributed (Supplementary Material Figure S8). The baseline characteristics of MetS according to weighted and unweighted lifestyle scores are presented in Supplementary Material Tables S4 and S5.

### 3.2. Associations of Single and Integrated Lifestyle Factors with All-Cause Mortality and Overall Cancer Incidence

**Figure 1:**
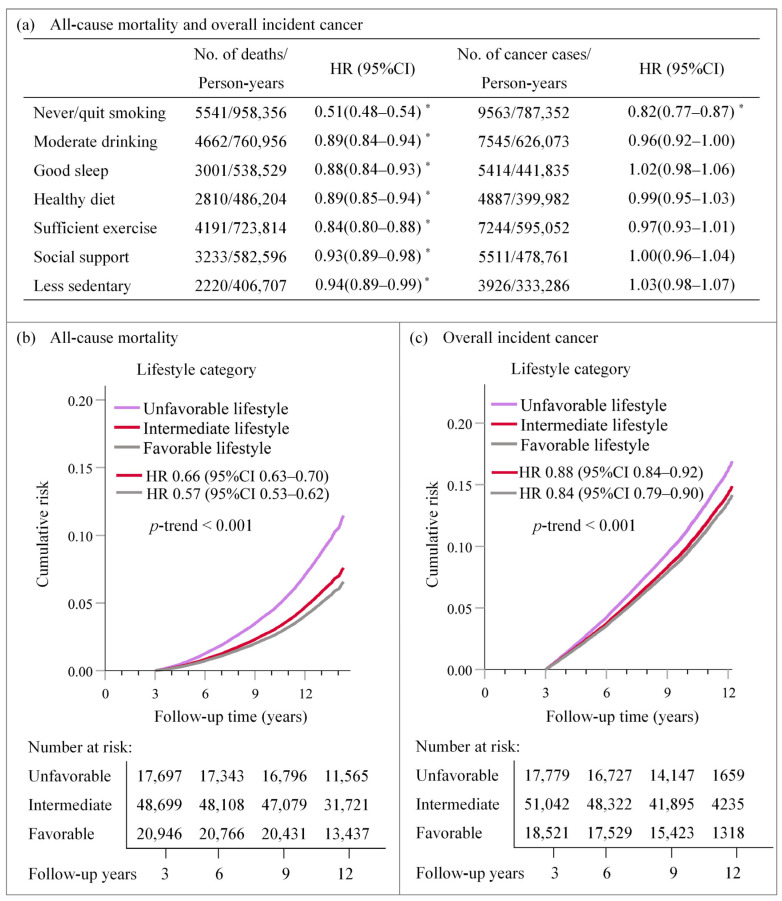
HR (95% CI) for all-cause mortality and overall incident cancer by individual and combined lifestyle factors. (**a**) HR (95% CI) of all-cause mortality and overall incident cancer according to individual lifestyle factors; (**b**) Cumulative risk and HR (95% CI) of all-cause mortality by lifestyle categories; (**c**) Cumulative risk and HR (95% CI) of overall incident cancer by lifestyle categories. Cox proportional hazards regression adjusted for age at baseline; sex; ethnicity; education; employment status; TDI; BMI category; medication treatment history for anti-cholesterol drug, antihypertensive drug, and insulin; family history of hypertension, DM, and cancer; and the number of MetS component traits. Part (**a**) considered the corresponding unrecommended behaviors as the reference group. Part (**b**,**c**) considered unfavorable lifestyles (lowest quintile of weighted lifestyle scores) as the reference group. Abbreviation: HR, Hazard ratio; CI, confidence interval; *, *p*-value < 0.05.

As shown in Figure 1, after total adjustment for potential confounders, never/quit smoking (HR: 0.51; 95% CI: 0.48–0.54), moderate alcohol consumption (HR: 0.89; 95% CI: 0.84–0.94), good sleep (HR: 0.88; 95% CI: 0.84–0.93), healthy diet (HR: 0.89; 95% CI: 0.85–0.94), sufficient exercise (HR: 0.84; 95% CI: 0.80–0.88), social support (HR: 0.93; 95% CI: 0.89–0.98), and less sedentary behaviour (HR: 0.94; 95% CI: 0.89–0.99) were all associated with a lower all-cause mortality risk compared to the corresponding unrecommended behaviour (*p*-value < 0.05). Never/quit smoking (HR: 0.82; 95% CI: 0.77–0.87) was associated with a lower risk of overall cancer incidence (Supplementary Material Table S6).

When integrating these seven factors, by considering each β lifestyle factor coefficient into a weighted lifestyle score and categorising them into three groups, it was found that participants with a favourable lifestyle (highest quintile) had HRs of 0.57 (95% CI: 0.53–0.62) for all-cause mortality and 0.84 (95% CI: 0.79–0.90) for overall cancer incidence compared to those with an unfavourable lifestyle (lowest quintile). Notably, a favourable lifestyle was associated with lower site-specific cancer risk in the digestive, respiratory, and intrathoracic organs (*p*-trend < 0.05) (Supplementary Material Table S7).

### 3.3. Associations of Healthy Lifestyle Scores with Cause-Specific Mortality

**Figure 2:**
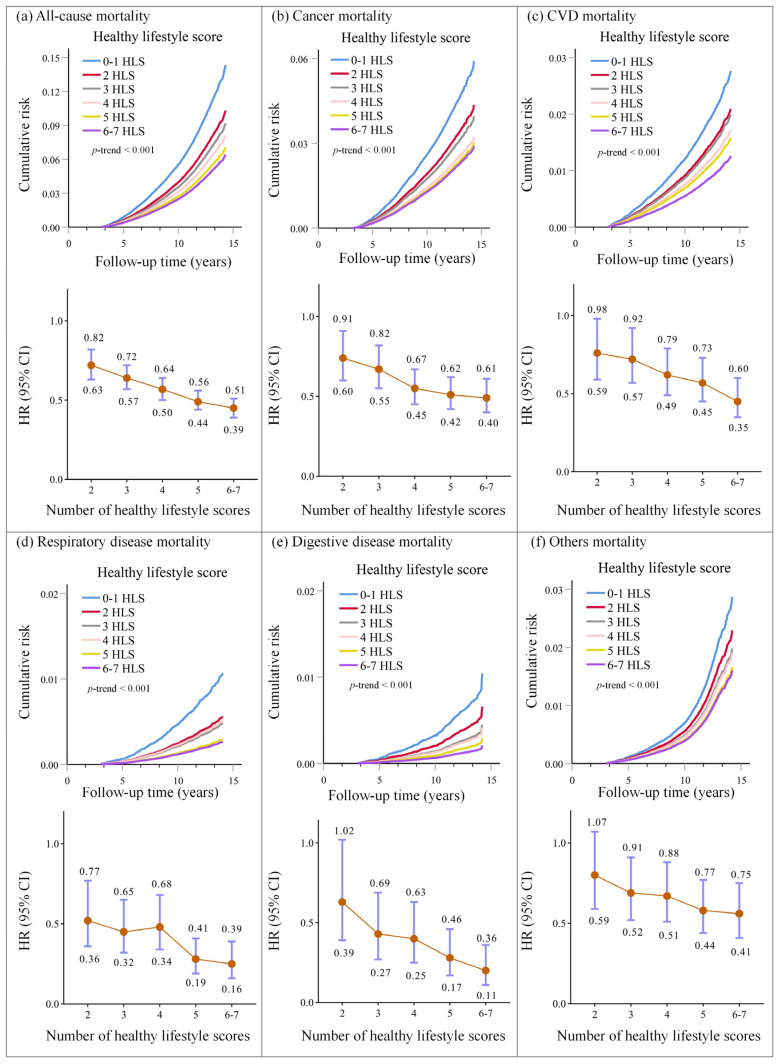
Cumulative risk and HR (95% CI) of all-cause and cause-specific mortality. Abbreviation: CVD, cardiovascular disease; HR, Hazard ratio; CI, confidence interval. Cox proportional hazards regression adjusted for age at baseline; sex; ethnicity; education; employment status; TDI; BMI categories; medication treatment history for anti-cholesterol drug, antihypertensive drug, and insulin; family history of hypertension, DM, and cancer; and the number of MetS component traits. 0–1 lifestyle score was considered the reference group.

**Figure 3:**
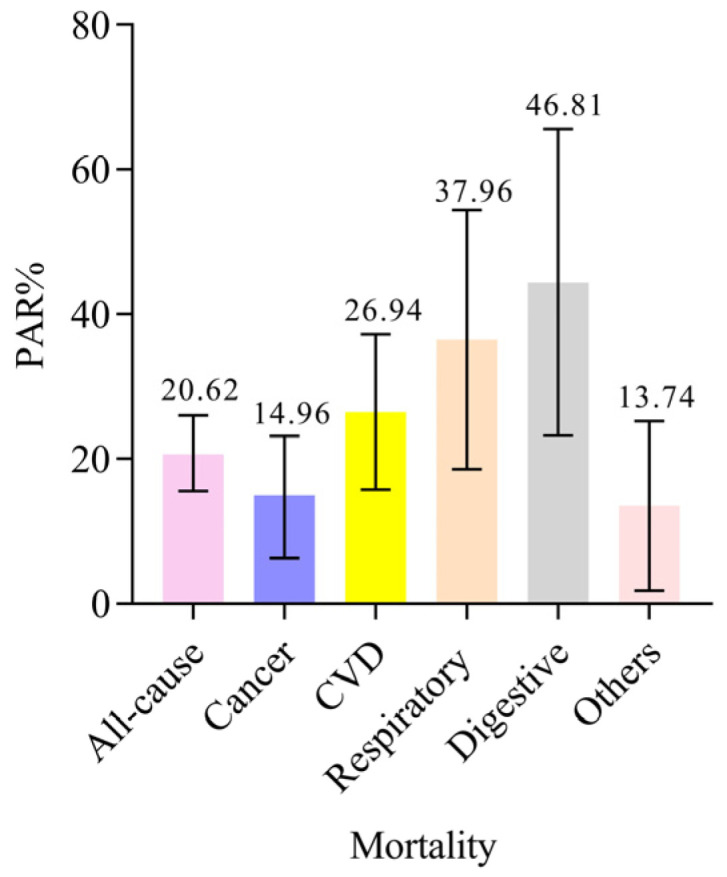
Multivariable-adjusted population-attributable risk percents (95% CI) for all-cause and cause-specific mortality. The PAR% of all-cause and cause-specific mortality is attributable to nonadherence to 6 or 7 recommended lifestyle behaviours. The multivariable model was adjusted for age at baseline; sex; ethnicity; education; employment status; TDI; BMI categories; medication treatment history for the anti-cholesterol drugs, antihypertensive drugs, and insulin; family history of hypertension, DM, and cancer; and the number of MetS component traits. Abbreviations: PAR%, population-attributable risk percent; CVD, cardiovascular disease.

Among 6739 deaths, 2712 (40.2%) were from cancer, 1731 (25.7%) from CVD, 535 (7.9%) from respiratory disease, 321 (4.8%) from digestive disease, and 1440 (21.4%) from all other causes. Based on the Cox proportional hazards regression curves of cumulative risk, all-cause mortality risk decreased monotonically across healthy lifestyle scores, and a higher healthy lifestyle score was associated with a lower risk of cause-specific mortality from respiratory disease, cancer, digestive disease, CVD, and other causes (*p*-value for trend < 0.001) (Figure 2 and Supplementary Material Table S8). The same pattern of effects was observed when lifestyle categories (weighted lifestyle scores) were used instead of lifestyle scores (Supplementary Material Table S9). The multivariable-adjusted PAR% (95% CI) suggested that 20.62% (15.57–26.03%) of all-cause mortality, 14.96% (6.32–23.18%) deaths from cancer, 26.94% (15.57–36.97%) deaths from CVD, 37.96% (17.89–53.63%) deaths from respiratory disease, and 46.81% (22.17–64.31%) deaths from the digestive disease were theoretically attributable to nonadherence to six or seven recommended lifestyle behaviours (Figure 3). Similar results were found in weighted lifestyle scores, where 18.57% of deaths were attributable to nonadherence to a favourable lifestyle (Supplementary Material Figure S9).

### 3.4. Subgroup Analysis by Baseline Characteristics

**Figure 4:**
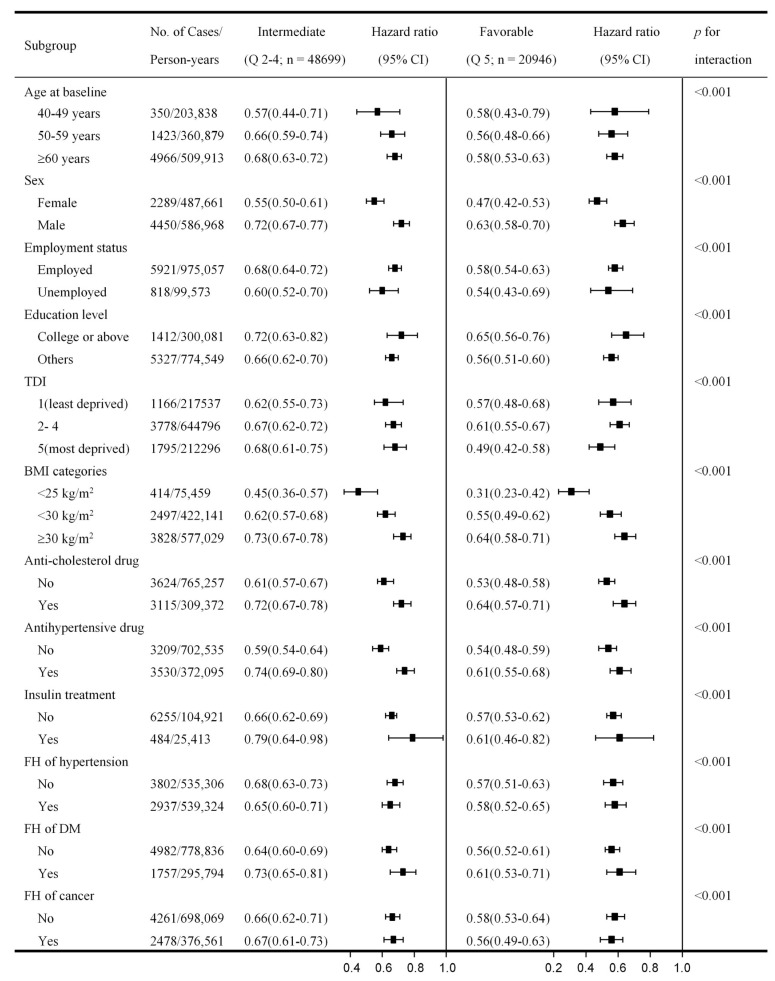
HR (95% CI) of all-cause mortality risk according to lifestyle categories stratified by baseline characteristics. Abbreviation: Q, quintiles; TDI, Townsend deprivation index; FH, family history; DM, diabetes mellitus. Cox proportional hazards regression adjusted for age at baseline; sex and ethnicity; education; employment status; TDI; BMI; medication treatment history for the anti-cholesterol drugs, antihypertensive drugs, and insulin; family history of hypertension, DM, and cancer; and the number of MetS component traits (excluded the corresponding variable when stratified by itself). The unfavorable lifestyle category (lowest quintile of weighted healthy lifestyle scores) was considered the reference group within each subgroup of baseline characteristics. All *p*-value for trend were <0.05. The *p* for interaction were estimated by including an interaction term of the corresponding baseline characteristic and lifestyle categories in the full model. The sum of Person-years may vary slightly due to the rounding.

Further analyses stratified by age at baseline, sex, employment status, education level, socioeconomic status, BMI categories, cholesterol medication, hypertension medication, insulin medication, family history of hypertension, DM, and cancer, with unfavourable lifestyle as the reference group, yielded a similar pattern of associations; favourable and intermediate lifestyles were inversely associated with all-cause mortality risk. Notably, crossover analysis also indicated that associations of favourable and intermediate lifestyles with all-cause mortality incidence were consistently stronger in participants with MetS who were women, employed, and had lower BMI (*p* for interaction < 0.001) (Figure 4 and Supplementary Material Table S10). Stronger associations for favourable and intermediate lifestyles were also identified in participants without a family history of DM (*p*-interaction < 0.001).

### 3.5. Sensitivity Analyses

Sensitivity analyses provided strong evidence for the robustness of the results. First, the unweighted lifestyle scores, which were categorised as scoring 0–2, 3–5, and 6–7, were inversely associated with all-cause and cause-specific mortality, as well as the overall and site-specific cancer risk (Supplementary Material Tables S11 and S12). Additionally, a similar pattern of results was observed when rebuilding the lifestyle categories by further including 18.5 ≤ BMI < 25 kg/m^2^ or redefining never smoking as a recommended behaviour (Supplementary Material Tables S13–S16).

## 4. Discussion

In this prospective cohort of 87,342 UK Biobank participants with baseline MetS, we found that a healthy lifestyle (integrating factors of never/quit smoking, moderate drinking, good sleep, healthy diet, sufficient exercise, social support, and less sedentary behaviour) was associated with a lower risk of all-cause and cause-specific mortality from cancer, respiratory diseases, digestive diseases, and CVD. Nearly one-fifth of deaths among populations with MetS could be attributed to nonadherence to six to seven recommended lifestyle behaviours. Notably, the contributing beneficial effect of adherence to six to seven health lifestyle behaviours to respiratory and digestive diseases is better than cancer and CVD diseases, possibly due to the other diseases, such as cancer, are more susceptible to genetic or other lifestyle factors [17]. Meanwhile, when participants with MetS had a favourable lifestyle, the risk of developing overall cancer incidence and site-specific cancer incidence in the digestive organs, breast, respiratory, and intrathoracic organs was reduced. Notably, there were significant interactions between lifestyle categories and baseline characteristics, such as age, sex, employment, and socioeconomic status, among others, on all-cause mortality.

Lifestyle factors affecting the risk of incident cancer and mortality have received widespread interest from researchers. A large cohort study with 444,399 members of the general population from the United States and the UK found that a healthy lifestyle (consisting of never smoking, moderate drinking, sufficient exercise, healthy diet) was associated with a reduced risk of all-cause mortality regardless of socioeconomic status [31]. Additionally, a previous study indicated the potential benefits of a healthy lifestyle (including not currently smoking, moderate drinking, adequate sleep duration, healthy diet, regular exercise, social support, and less sedentary behaviour) in reducing the risk of all-cause mortality and mortality from cancer, CVD, respiratory and digestive disease among T2DM individuals [38]. Our study confirmed a similar trend in mortality among individuals with MetS, although the definitions of a healthy lifestyle varied slightly. These findings emphasise the importance of lifestyle management to prevent premature death and extend healthy life expectancy in adults with MetS. More benefits were shown among women, unemployed participants with lower socioeconomic status, lower BMI, and a family history of cancer.

Additionally, a previous study among Swedish women found that quitting smoking and weight reduction were inversely associated with the risk of cancer, including breast cancer (HR < 1; *p* < 0.05) [39]. Moreover, a previous study from Chile provided evidence that eliminating lifestyle risk factors of insufficient physical activity, smoking, drinking, high BMI, and low intake of fruits and vegetables reduced approximately half of the respiratory and digestive cancer cases [40]. These findings are consistent with the results of this study. Our results add to the current literature by validating the fact that a favourable lifestyle is correlated with a lower cancer risk among adults with MetS. These findings highlight the potential of modifiable lifestyle factors for cancer prevention in populations with MetS, as well as on site-specific cancer incidence in the digestive, respiratory, and intrathoracic organs.

To our knowledge, no previous study has examined the associations between lifestyle factors, cancer incidence, and mortality risk among adults with MetS. Two recent systematic reviews and meta-analyses found that lifestyle interventions improve the risk factors and prognosis of MetS [12,41]. However, these studies had lifestyle interventions limited to a single diet and physical activity factors. Only six studies combined four lifestyle factors (smoking, drinking, exercise, and diet) [31,42,43,44,45,46]. In addition, mortality and cancer risk as health outcomes of interest have not been studied. Compared to previous studies, we used data from populations with MetS in order of magnitude larger, which guaranteed us to use sufficient statistical power for joint and stratified analyses and examine multiple health outcomes of interest.

Multiple mechanisms were proposed to explain the associations between lifestyle factors, all-cause, cause-specific mortality, and cancer incidence. The release of carcinogens from tobacco products, including tobacco-specific nitrosamines and volatile organic compounds, causes complex mutations in key cancer genes [47], and pro-lung inflammatory and gut microbial derangement effects of tobacco smoke have also been identified [48,49]. Alcohol consumption is known to have a negative effect on vitamins, such as B6, and multivitamin deficiency may lead to protein and energy malnutrition, which is linked to cancer development [50]. Moreover, excessive consumption of alcohol produces acetaldehyde [51], contributes to the induction of oxidative stress [52], hinders folate absorption, increases inflammation, and decreases immune function [53,54]. Acetaldehyde can also accumulate in the gastrointestinal tract and damage the gastrointestinal mucosa [55]. Additionally, excessive drinking may reduce the ability of immune cells to defend against bacteria in the lungs, increasing susceptibility to respiratory infections [56]. Sleep disorders may lead to abnormal expression of circadian clock genes, interruption of melatonin release, decreased immunity, exacerbated gastroesophageal reflux disease, and increased hypersensitivity of esophageal [57,58]. A healthy diet, such as the Mediterranean diet, is known for its lipid-lowering effect, antioxidant and anti-inflammatory abilities, platelet aggregation, gut homeostasis maintenance, lung function protection, and modification of growth factors and hormones involved in cancer pathogenesis, which is beneficial to health and longevity [59,60,61]. Sufficient physical activity is widely believed to improve immune function, delay aging, promote DNA repair, and extend lifespan [62,63]. Sedentary behaviour is often linked to obesity [64]. Social support contributes to health and longevity, as bonding with partners is beneficial for eliminating or attenuating the adverse consequences of the hypothalamic–pituitary–adrenocortical axis and the activation of the sympathetic–adreno –medullary axis [65].

This study has several limitations. First, lifestyle information was collected through self-reports and only investigated once, which may have caused measurement errors. Lifestyles are not static, and repeated measures should be considered in future studies. Second, although a weighted lifestyle score was created, with the assumption that each factor has a different effect on mortality and cancer, to examine the effect of healthy lifestyle factors on the outcomes, it was still difficult to fully explain the complex interactions between lifestyle factors, and the combined lifestyle factors did not answer the question about the different factors with diverting effects to complex diseases. Third, a limited number of lifestyle factors were included in this study, and other environmental and lifestyle factors may also have an effect on cancer or death risk. Fourth, although the study had adjusted for potential confounders and participants without cancer at baseline were followed up for a median of approximately 12 years, there may still be unmeasured confounders and reverse causality. Fifth, the participants in this study were primarily Caucasians, with a baseline age of 40–70 years. Further research on other ethnic groups is required. Finally, due to the limitations of observational studies, it is difficult to derive causality; well-designed interventional studies are warranted in the near future.

## 5. Conclusions

This study, which is based on a large, nationwide UK Biobank cohort, suggests that adherence to a favourable lifestyle is significantly associated with a lower risk of mortality and incident cancer among populations with MetS. These findings highlight the importance of lifestyle modifications in reducing the disease burden for individuals with MetS. However, whether the observed associations are causal remains to be further determined.

## Figures and Tables

**Table 1:** Baseline characteristics of participants by all-cause mortality and overall cancer incidence.

Characteristic	All-Cause Mortality		Overall Cancer Incidence	
	No (*n* = 80,603)	Yes (*n* = 6739)	No (*n* = 76,540)	Yes (*n* = 10,802)
Age, mean (SD), years ^t^	57.2 (7.7)	62.0 (6.1)	57.1 (7.8)	60.8 (6.3)
Male, *n* (%) *	43,384 (53.8)	4450 (66.4)	41,261 (53.9)	6573 (60.8)
BMI, mean (SD) ^t^	30.9 (4.7)	31.5 (5.1)	31.0 (4.8)	30.8 (4.5)
White Ethnicity, *n* (%) *	74,642 (92.6)	6397 (94.9)	70,615 (92.3)	10,424 (96.5)
Employed, *n* (%) *	73,219 (90.8)	5921 (87.9)	69,163 (90.4)	9977 (92.4)
College/university, *n* (%) *	22,862 (28.4)	1412 (21.0)	21,540 (28.1)	2734 (25.3)
TDI, *n* (%) ^w^				
1 (least deprived)	16,314 (20.2)	1166 (17.3)	15,152 (19.8)	2328 (21.6)
2–4	48,617 (60.3)	3778 (56.1)	45,850 (59.9)	6545 (60.6)
5 (most deprived)	15,672 (19.4)	1795 (26.6)	15,538 (20.3)	1929 (17.9)
Medication, *n* (%) *				
Anti-cholesterol drug	22,453 (27.9)	3115 (46.2)	21,750 (28.4)	3818 (35.3)
Antihypertensive drug	27,080 (33.6)	3530 (52.4)	26,108 (34.1)	4502 (41.7)
Insulin	1698 (2.1)	484 (7.2)	1906 (2.5)	276 (2.6)
Family history, *n* (%) *				
Hypertension	40,801 (49.4)	2937 (43.6)	38,709 (50.6)	5029 (46.6)
DM	22,375 (27.8)	1757 (26.1)	21,424 (28.0)	2708 (25.1)
Cancer	28,158 (34.9)	2478 (36.8)	26,366 (34.4)	4270 (39.5)
No. of MetS components, *n* (%) ^w^				
3	53,232 (66.0)	3964 (58.8)	50,230 (65.6)	6966 (64.5)
4	22,022 (27.3)	2061 (30.6)	21,084 (27.5)	2999 (27.8)
5	5349 (6.6)	714 (10.6)	5226 (6.8)	837 (7.7)
Healthy lifestyle factor, *n* (%) *				
Never/quit smoking	72,234 (89.6)	5541 (82.2)	68,212 (89.1)	9563 (88.5)
Moderate drinking	57,421 (71.2)	4662 (69.2)	54,538 (71.3)	7545 (69.8)
Good sleep	40,625 (50.4)	3001 (44.5)	38,212 (49.9)	5414 (50.1)
Healthy diet	36,708 (45.4)	2810 (41.7)	34,631 (45.2)	4887 (45.2)
Sufficient exercise	54,578 (67.7)	4191 (62.2)	51,525 (67.3)	7244 (67.1)
Social support	43,990 (54.6)	3233 (48.0)	41,712 (54.5)	5511 (51.0)
Less sedentary	30,663 (38.0)	2220 (32.9)	28,957 (37.8)	3926 (36.3)

Abbreviations: BMI, body mass index; TDI, Townsend deprivation index; DM, diabetes mellitus; MetS, metabolic syndrome; SD, standard deviation; *, the binary variable shows the percentage of one of the columns. All *p*-values were < 0.05 except for the distribution between insulin treatment, lifestyle factors of smoking, sleep, diet, and social support with overall cancer incidence. Pearson’s χ^2^ test was used to analyse all variables except for those indicated; ^t^ means Student’s *t*-test and ^w^ means Wilcoxon rank test. Due to rounding, the column percentage sum may not be 100%.

## Data Availability

The UK Biobank datasets are openly available by submitting a data request proposal from https://www.ukbiobank.ac.uk/ (accessed on 9 April 2022). We are authorised to access the database through the Access Management System (AMS) (Application number: 78563).

## Supplementary Materials

The following supporting information can be downloaded at: https://www.mdpi.com/article/10.3390/ijerph19169936/s1, Table S1. Healthy lifestyle behavior definitions; Table S2. Coding of outcomes; Table S3. Characteristics of MetS participants included or excluded from the current study; Table S4. Baseline characteristics of MetS participants by weighted lifestyle scores; Table S5. Baseline characteristics of MetS participants by unweighted lifestyle scores; Table S6. HR (95% CI) for all-cause mortality and overall cancer incidence by individual lifestyle factors; Table S7. HR (95% CI) for overall and site-specific cancer by weighted lifestyle categories; Table S8. HR (95% CI) for all-cause and cause-specific mortality by unweighted healthy lifestyle scores; Table S9. HR (95% CI) for all-cause and cause-specific mortality by weighted lifestyle categories; Table S10. Crossover analysis of interaction between lifestyle categories (weighted lifestyle scores) and baseline characteristics on all-cause mortality risk; Table S11. Association of unweighted lifestyle scores category with all-cause and cause-specific mortality risk; Table S12. Association of unweighted lifestyle scores category with overall and site-specific cancer risk; Table S13. HR (95% CI) of all-cause and cause-specific mortality risk according to unweighted lifestyle category with further inclusion of BMI; Table S14. HR (95% CI) of overall and site-specific cancer risk according to unweighted lifestyle category with further inclusion of BMI; Table S15. HR (95% CI) of all-cause and cause-specific mortality risk according to unweighted lifestyle category with further redefining never smoke as a healthy behavior; Table S16. HR (95% CI) of overall and site-specific cancer risk according to unweighted lifestyle category with further redefining never smoke as a healthy behavior; Figure S1. Cohort exclusions of the study participants; Figure S2. Schoenfeld residuals test for all-cause mortality; Figure S3. Schoenfeld residuals test for cancer mortality; Figure S4. Schoenfeld residuals test for cardiovascular disease mortality; Figure S5. Schoenfeld residuals test for respiratory disease mortality; Figure S6. Schoenfeld residuals test for digestive disease mortality; Figure S7. Schoenfeld residuals test for overall incident cancer; Figure S8. Distribution of the healthy lifestyle scores; Figure S9. Multivariable-adjusted population-attributable risk percents (95% CI) for all-cause and cause-specific mortality according to weighted lifestyle scores.

## Abbreviations

BMI: body mass index; BP: blood pressure; HRs: hazard ratios; CIs: confidence intervals; CVD: cardiovascular disease; T2DM: type 2 diabetes mellitus; DM: diabetes mellitus; FBG: fasting blood glucose; HDL-C: high-density lipoprotein cholesterol; TG: triglycerides; HRs: hazard ratios; ICD: International Classification of Diseases; MetS: Metabolic syndrome; PAR: population-attributable risk; TDI: Townsend deprivation index.
