# Supplementary material for: Association of a Healthy Lifestyle with All-Cause, Cause-Specific Mortality and Incident Cancer among Individuals with Metabolic Syndrome: A Prospective Cohort Study in UK Biobank
Source: Int J Environ Res Public Health. 2022 Aug 11;19(16):9936. doi: 10.3390/ijerph19169936 (PMC9408492; doi:10.3390/ijerph19169936)
Supplement: Supplementary file 1 [file ijerph-19-09936-s001.zip › ijerph-1795962-supplementary.pdf]

Supplementary Materials:

Table S1. Healthy lifestyle behavior definitions

Table S2. Coding of outcomes

Table S3. Characteristics of MetS participants included or excluded from the current study

Table S4. Baseline characteristics of MetS participants by weighted lifestyle scores

Table S5. Baseline characteristics of MetS participants by unweighted lifestyle scores

Table S6. HR (95% CI) for all-cause mortality and overall cancer incidence by individual lifestyle factors

Table S7. HR (95% CI) for overall and site-specific cancer by weighted lifestyle categories

Table S8. HR (95% CI) for all-cause and cause-specific mortality by unweighted healthy lifestyle scores

Table S9. HR (95% CI) for all-cause and cause-specific mortality by weighted lifestyle categories

Table S10. Crossover analysis of interaction between lifestyle categories (weighted lifestyle scores) and baseline characteristics on all-cause mortality risk

Table S11. Association of unweighted lifestyle scores category with all-cause and cause-specific mortality risk

Table S12. Association of unweighted lifestyle scores category with overall and site-specific cancer risk

Table S13. HR (95% CI) of all-cause and cause-specific mortality risk according to unweighted lifestyle category with further inclusion of BMI

Table S14. HR (95% CI) of overall and site-specific cancer risk according to unweighted lifestyle category with further inclusion of BMI

Table S15. HR (95% CI) of all-cause and cause-specific mortality risk according to unweighted lifestyle category with further redefining never smoke as a healthy behavior

Table S16. HR (95% CI) of overall and site-specific cancer risk according to unweighted lifestyle category with further redefining never smoke as a healthy behavior

Figure. S1 Cohort exclusions of the study participants

Figure. S2 Schoenfeld residuals test for all-cause mortality

Figure. S3 Schoenfeld residuals test for cancer mortality

Figure. S4 Schoenfeld residuals test for cardiovascular disease mortality

Figure. S5 Schoenfeld residuals test for respiratory disease mortality

Figure. S6 Schoenfeld residuals test for digestive disease mortality

Figure. S7 Schoenfeld residuals test for overall incident cancer

Figure. S8 Distribution of the healthy lifestyle scores

Figure. S9 Multivariable-adjusted population-attributable risk percents (95% CI) for all-cause and cause-specific mortality according to weighted lifestyle scores

Table S1. Healthy lifestyle behavior definitions

Healthy lifestyle behavior	Field IDs	Healthy lifestyle data-coding
Never/quit-smoking	20116	0. Never. 1. Previous.
Alcohol consumption ≤ 14/28g/day for women/men	1568,4407 1578, 4418	125ml red wine ≈0.85 servings.<br>125ml white wine ≈ 0.85 servings.
	1588, 4429	568 ml beer ≈1.28 servings.
	1598, 4440	25ml spirits ≈ 0.57 servings.
	1608, 4451	50ml fortified wine ≈ 0.56 servings.
	4462, 5364	125ml alcopops ≈ 0.36 servings.
		* 1 serving was defined as 14g.
Good sleep	1160	7–8
	1210	2.No
Diet behaviors ≥ 4 groups	1309, 1319	Fruit ≥ 3 pieces/day
	1289, 1299	Vegetables ≥ 3 tablespoons/day
	1329, 1339	Fish ≥ twice a week
	1438, 1448	Whole grains ≥ 3 slices/day
	1349	Processed meat ≤ once a week.
	1369,1379,1389	Unprocessed meat ≥ twice a week
	1458, 1468	Refined grains ≤ 1 servings/day
Sufficient physical activity	884	at least 5 days a week
	894	≥150 minutes moderate activity per week
	904	at least once a week
	914	≥75 minutes moderate activity per week
		* Meet any of the above or an equivalent combination
Social support	1031	1.Almost daily. 2.2-4 times a week.<br>3. About once a week
	2110	3. About once a week. 4.2-4 times a week<br>5.Almost daily
	6141	Had people in household related to
	6160	Had leisure/social activities
		* Containing at least 2 of the social connections above.
Sedentary behavior ≤ 4 hours/day	1070	Time spent watching TV
	1080	Time spent using computer
	1090	Time spent driving
		* TV + computer + driving ≤ 4 h/day

Table S2. Coding of outcomes

Outcome	ICD-10	ICD-9	Self-reported
Cause of death			
Cancer	C00-C97		
CVD	I05-I89		
Respiratory disease	J09-J99		
Digestive disease	K20-K93		
Over all cancer	C00-C97	140-208	(-1) to 9999
Site-specific cancer			
Digestive organs	C15-C26	150-159	1017-1023
Respiratory and intrathoracic organs	C30-C39	160-165	1001, 1006-1009, 1027-1028,1080-1084
Breast	C50-C50	174-175	1002

[Abbreviation: CVD, cardiovascular disease. ICD, International Classification of Disease.]

Table S3. Characteristics of MetS participants included or excluded from the current study

Characteristics	Over all MetS	Included	Excluded
No. of participants	137591	87342	50249
Age, years *	60 [52, 60]	59 [52,64]	60 [54, 65]
Sex			
Female	68700 (49.9)	39508 (45.2)	29192 (58.1)
Male	68891 (50.1)	47834 (54.8)	21057 (41.9)
BMI, kg/m^2 *	30.5 [27.9,33.6]	30.4 [27.8, 33.4]	30.7 [27.9,34.2]
Ethnicity			
White	127443 (92.6)	81039 (92.8)	46404 (92.3)
Mixed	1963 (1.4)	1219 (1.4)	744 (1.5)
Asian	5370 (3.9)	3604 (4.1)	1766 (3.5)
Black	2371 (1.7)	1480 (1.7)	891 (1.8)
Missing value	444 (0.3)		444 (0.3)
Townsend deprivation index *	-1.8 [(-3.5), 1.1]	-1.9 [(-3.5),0.8]	-1.5 [(-3.3),1.7]
Employment			
Employed	122798 (89.2)	79140 (90.6)	43658 (13.1)
Others	14793 (10.8)	8202 (9.4)	6591 (13.1)
Education			
College/university	34897 (25.4)	24274 (27.8)	10623 (21.1)
Others	102694 (74.6)	63068 (72.2)	39626 (78.9)
Medication, n (%) +			
Anti-cholesterol drug	41511 (30.2)	25568 (29.3)	15943 (31.7)
Antihypertensive drug	50198 (36.5)	30610 (35.0)	19588 (39.0)
Insulin	3902 (2.8)	2182 (2.5)	1720 (3.4)
Family history +			
Hypertension	68271 (49.6)	43738 (50.1)	24533 (48.8)
DM	38289 (27.8)	24132 (27.6)	14157 (28.2)
Cancer	48812 (35.5)	30636 (35.1)	18176 (36.2)

{Abbreviations: BMI, body mass index; DM, diabetes mellitus; MetS, metabolic syndrome; +, the binary variable shows the percentage of one of the columns. *, Continuous variables were presented as median [interquartile range].

Categorical variables were presented as frequencies (%).

Participants with cancers at baseline, lost to follow-up, with age < 40 years, with missing information, who died or had cancer within the first three years after recruitment were excluded.}

Table S4. Baseline characteristics of MetS participants by weighted lifestyle scores

Characteristic	Weighted lifestyle scores for mortality			Weighted lifestyle scores for overall cancer incidence		
	Unfavorable (Quintile 1; n = 17697)	Intermedium (Quintile 2-4; n = 48699)	Favorable (Quintile 5; n = 20946)	Unfavorable (Quintile 1; n = 17779)	Intermedium (Quintile 2-4; n = 51042)	Favorable (Quintile 5; n = 18521)
Age, n (%)						
40-49 years	3811(21.5)	8356(17.2)	4097(19.6)	4304(24.2)	9157(17.9)	2803(15.1)
50-59 years	6240(35.3)	16134(33.1)	6646(31.7)	6457(36.3)	16899(33.1)	5664(30.6)
≥ 60 years	7646(43.2)	24209(49.7)	10203(48.7)	718(39.5)	24986(49.0)	10054(54.3)
Sex, n (%)						
Female	6601(37.3)	22153(45.5)	10754(51.3)	7147(40.2)	23717(46.5)	8644(46.7)
Male	11096(62.7)	26546(54.5)	10192(48.7)	10632(59.8)	27325(53.5)	9877(53.3)
Ethnicity, n (%)						
White	16705(94.4)	45317(93.1)	19017(90.8)	16811(94.6)	47586(93.2)	16642(89.9)
Mixed	247(1.4)	646(1.3)	326(1.6)	243(1.4)	622(1.2)	354(1.4)
Asian	466(2.6)	1889(3.9)	1248(6.0)	498(2.8)	2074(4.1)	1032(5.6)
Black	279(1.6)	847(1.7)	354(1.7)	227(1.3)	760(1.5)	493(2.7)
TDI, n (%)						
1 (least deprived)	2845(16.1)	10023(20.6)	4612(22.0)	3055(17.2)	10621(20.8)	3804(20.5)
2- 4	9986(56.4)	29530(60.6)	12879(61.5)	10048(56.5)	31087(60.9)	11260(60.8)
5 (most deprived)	4866(27.5)	9146(18,8)	3455(16.5)	4676(26.3)	9334(18.3)	3457(18.7)
Employment, n (%)						
Employed	15161(85.7)	44469(91.3)	19510(93.1)	15540(87.4)	46596(91.3)	17004(91.8)
Unemployed	2536(14.3)	4230(8.7)	1436(6.9)	2239(12.6)	4446(8.7)	1517(8.2)
Education, n (%)						
College/university	3963(22.4)	13239(27.2)	7072(33.8)	4667(26.3)	24608(28.6)	4999(27.0)
Others	13734(77.6)	35460(72.8)	13874(66.2)	13112(73.7)	36434(71.4)	13522(73.0)
BMI, n (%)						

< 25 kg/m^2	1179(6.7)	2987(6.1)	1973(9.4)	1348(7.6)	3529(6.9)	1262(6.8)
25-30 kg/m^2	6778(38.3)	18504(38.0)	8962(42.8)	7284(41.0)	19772(38.7)	7187(38.8)
≥ 30 kg/m2	9740(27208)	27208(55.9)	10011(47.8)	9147(51.4)	27740(54.3)	10072(54.4)
Anti-cholesterol drug, n (%)						
No	12154(68.7)	34021(69.9)	15599(74.5)	12820(72.1)	36321(71.2)	12633(68.2)
Yes	5543(31.3)	14678(30.1)	5347(25.5)	4959(27.9)	14721(28.8)	5888(31.8)
Antihypertensive drug, n (%)						
No	11475(64.8)	30978(63.6)	14279(68.2)	12099(68.1)	32988(64.6)	11645(62.9)
Yes	6222(35.2)	17721(36.4)	6667(31.8)	5680(31.9)	18054(35.4)	6876(37.1)
Insulin, n (%)						
No	17199(97.2)	47452(97.4)	20509(97.9)	17375(97.7)	49783(97.5)	18002(97.2)
Yes	498(2.8)	1247(2.6)	437(2.1)	404(2.3)	1259(2.5)	519(2.8)
Family history of hypertension, n (%)						
No	9325(52.7)	24061(49.4)	10218(48.8)	9235(51.9)	25254(49.5)	9115(49.2)
Yes	8372(47.3)	24638(50.6)	10728(51.2)	8544(48.1)	25788(50.5)	9406(50.8)
Family history of DM, n (%)						
No	12959(73.2)	35211(72.3)	15040(71.8)	13176(74.1)	36880(72.3)	13154(71.0)
Yes	4738(26.8)	13488(27.7)	5906(28.2)	4603(25.9)	14162(27.7)	5367(29.0)
Cancer, n (%)						
No	11469(64.8)	31286(64.2)	13951(66.6)	11681(65.7)	33007(64.7)	12018(64.9)
Yes	6228(35.2)	17413(35.8)	6995(33.4)	6098(34.3)	18035(35.3)	6503(35.1)
No. of MeTs components, n (%)						
3	11391(64.4)	31557(64.8)	14248(68.0)	11816(66.5)	33547(65.7)	11833(63.9)
4	5002(28.3)	13673(28.1)	5408(25.8)	4853(27.3)	13977(27.4)	5253(28.4)
5	1304(7.4)	3469(7.1)	1290(6.2)	1110(6.2)	3518(6.9)	1435(7.7)

[Abbreviations: TDI, Townsend deprivation index; BMI, body mass index; DM, diabetes mellitus; MetS, metabolic syndrome.]

Table S5. Baseline characteristics of MetS participants by unweighted lifestyle scores

	Unweighted lifestyle scores					
Characteristic	0-1 (n = 2243)	2 (n = 7875)	3 (n = 17408)	4 (n = 24083)	5 (n = 21609)	6-7 (n = 14124)
Age, n (%)						
40-49 years	475(21.2)	1425(18.1)	3190(18.3)	4325(18.0)	4130(19.0)	2746(19.4)
50-59 years	813(36.2)	2757(35.0)	5921(34.0)	7980(33.1)	7034(32.6)	4515(32.0)
≥ 60 years	955(42.6)	3693(46.9)	8297(47.7)	11778(48.9)	10472(48.5)	6863(48.6)
Sex, n (%)						
Female	630(28.1)	2720(34.5)	6749(38.8)	10560(43.8)	10688(49.5)	8161(57.8)
Male	1613(71.9)	5155(65.5)	10659(61.2)	13523(56.2)	10921(50.5)	5963(42.2)
Ethnicity, n (%)						
White	2166(96.6)	7567(96.1)	16531(94.9)	22398(93.0)	19727(91.3)	12668(89.7)
Mixed	18(0.8)	83(1.1)	197(1.1)	319(1.3)	360(1.7)	242(1.7)
Asian	28(1.2)	128(1.6)	428(2.5)	905(3.8)	1142(5.3)	973(6.9)
Black	31(1.4)	97(1.2)	270(1.6)	461(1.9)	380(1.8)	241(1.7)
TDI, n (%)						
1 (least deprived)	315(14.0)	1371(17.4)	3253(18.7)	4965(20.6)	4553(21.1)	3024(21.4)
2- 4	1245(55.5)	4665(59.2)	10336(59.4)	14353(59.6)	13130(60.8)	8666(61.4)
5 (most deprived)	683(30.5)	1839(23.4)	3820(21.9)	4765(19.8)	3926(18.2)	2434(17.2)
Employment, n (%)						
Employed	1822(81.2)	6822(86.6)	15468(88.9)	21983(91.3)	19924(92.2)	13120(92.9)
Unemployed	421(18.8)	1053(13.4)	1939(11.1)	2100(8.7)	1685(7.8)	1004(7.1)
Education, n (%)						
College/university	400(17.8)	1681(21.3)	4029(23.1)	6448(26.8)	6606(30.6)	5110(36.2)
Others	1843(82.2)	6194(78.7)	13379(76.9)	17635(73.2)	15003(69.4)	9014(63.8)
BMI, n (%)						

< 25 kg/m^2	133(5.9)	362(4.6)	868(5.0)	1527(6.3)	1699(7.9)	1550(11.0)
25-30 kg/m^2	784(35.0)	2784(35.4)	6307(36.2)	9367(38.9)	8801(40.7)	6201(43.9)
≥ 30 kg/m2	1326(59.1)	4729(60.1)	10233(58.8)	13189(54.8)	11109(51.4)	6373(45.1)
Anti-cholesterol drug, n (%)						
No	1495(66.7)	5190(65.9)	11796(67.8)	16872(70.1)	15734(72.8)	10687(75.7)
Yes	748(33.3)	2685(34.1)	5612(32.2)	7211(29.9)	5875(27.2)	3437(24.3)
Antihypertensive drug, n (%)						
No	1403(62.6)	4773(60.6)	10851(62.3)	15611(64.8)	14311(66.2)	9783(69.3)
Yes	840(37.4)	3102(39.4)	6557(37.7)	8472(35.2)	7298(33.8)	4341(30.7)
Insulin, n (%)						
No	2188(97.5)	7635(97.0)	16931(97.3)	23450(97.4)	21126(97.8)	13830(97.9)
Yes	55(2.5)	240(3.0)	477(2.7)	633(2.6)	483(2.2)	294(2.1)
Family history of hypertension, n (%)						
No	1207(53.8)	4080(51.8)	8857(50.9)	12071(50.1)	10544(48.8)	6845(48.5)
Yes	1036(46.2)	3795(48.2)	8551(49.1)	12012(49.9)	11065(51.2)	7279(51.5)
Family history of DM, n (%)						
No	1637(73.0)	5813(73.8)	12667(72.8)	17479(72.6)	15459(71.5)	10155(71.9)
Yes	606(27.0)	2062(26.2)	4741(27.2)	6604(27.4)	6150(28.5)	3969(28.1)
Cancer, n (%)						
No	1401(62.5)	4951(62.9)	11102(63.8)	15652(65.0)	14120(65.3)	9480(67.1)
Yes	842(37.5)	2924(37.1)	6306(36.2)	8431(35.0)	7489(34.7)	4644(32.9)
No. of MeTs components, n (%)						
3	1451(64.7)	5071(64.4)	11114(63.8)	15570(64.7)	14321(66.3)	9669(68.5)
4	658(29.3)	2167(27.5)	4982(28.6)	6820(28.3)	5829(27.0)	3627(25.7)
5	134(6.0)	637(8.1)	1312(7.5)	1693(7.0)	1459(6.8)	828(5.9)

[Abbreviations: TDI, Townsend deprivation index; BMI, body mass index; DM, diabetes mellitus; MetS, metabolic syndrome.]

Table S6. HR (95% CI) for all-cause mortality and overall cancer incidence by individual lifestyle factors

	All-cause mortality	*p*-vlaue	Overall cancer	*p*-vlaue
Never/quit smoking				
Model 1	0.47(0.44-0.50)	<0.001	0.82(0.77-0.87)	<0.001
Model 2	0.52(0.49-0.56)	<0.001	0.82(0.77-0.87)	<0.001
Model 3	0.51(0.48-0.54)	<0.001	0.82(0.77-0.87)	<0.001
Moderate drinking				
Model 1	0.92(0.87-0.97)	0.001	0.96(0.92-1.00)	0.067
Model 2	0.92(0.87-0.97)	0.001	0.96(0.92-1.00)	0.060
Model 3	0.89(0.84-0.94)	<0.001	0.96(0.92-1.00)	0.057
Good sleeping				
Model 1	0.80(0.76-0.84)	<0.001	1.01(0.97-1.05)	0.537
Model 2	0.85(0.81-0.90)	<0.001	1.02(0.98-1.06)	0.417
Model 3	0.88(0.84-0.93)	<0.001	1.02(0.98-1.06)	0.310
Healthy diet				
Model 1	0.86(0.82-0.90)	<0.001	0.99(0.95-1.03)	0.471
Model 2	0.89(0.85-0.93)	<0.001	0.99(0.95-1.03)	0.519
Model 3	0.89(0.85-0.94)	<0.001	0.99(0.95-1.03)	0.552
Sufficient exercise				
Model 1	0.77(0.73-0.81)	<0.001	0.96(0.92-1.00)	0.054
Model 2	0.80(0.77-0.84)	<0.001	0.96(0.93-1.00)	0.073
Model 3	0.84(0.80-0.88)	<0.001	0.97(0.93-1.01)	0.111
Social support well				
Model 1	0.93(0.89-0.98)	0.004	1.00(0.96-1.04)	0.981
Model 2	0.93(0.89-0.98)	0.004	1.00(0.96-1.04)	0.997
Model 3	0.93(0.89-0.98)	0.004	1.00(0.96-1.04)	0.966
Less sedentary behavior				
Model 1	0.90(0.85-0.94)	<0.001	1.02(0.98-1.06)	0.354
Model 2	0.91(0.86-0.96)	<0.001	1.02(0.98-1.06)	0.326
Model 3	0.94(0.89-0.99)	0.022	1.03(0.98-1.07)	0.241

[Model 1: adjusted for age at baseline, sex and ethnicity.

Model 2: Model 1 plus education, employment status and TDI.

Model 3: Model 2 plus BMI; medication treatment history for anti-cholesterol drug, antihypertensive drug, and insulin; family history of hypertension, DM, cancer; and the number of MetS component traits.]

Table S7. HR (95% CI) for overall and site-specific cancer by weighted lifestyle categories

Endpoints	Lifestyle category (weighted lifestyle scores for cancer)		
	Unfavorable (Quintile 1; n = 17779)	Intermedium (Quintile 2-4; n = 51042)	Favorable (Quintile 5; n = 18521)
Overall cancer (n = 10802)			
No. of cases/person-years	2272/179151	6280/516912	2250/186909
HR (95% CI)	1.00 (ref)	0.88(0.84-0.92)	0.84(0.79-0.90)
p-value		<0.001	<0.001
p-value for trend	<0.001		
Malignant neoplasms of digestive organs (n = 1665)			
No. of cases/person-years	379/179151	952/516912	334/186909
HR (95% CI)	1.00 (ref)	0.82(0.73-0.93)	0.75(0.65-0.88)
p-value		0.001	<0.001
p-value for trend	<0.001		
Malignant neoplasms of respiratory and intrathoracic organs (n = 675)			
No. of cases/person-years	289/179151	286/516912	100/186909
HR (95% CI)	1.00 (ref)	0.33(0.28-0.39)	0.29(0.23-0.37)
p-value		<0.001	<0.001
p-value for trend	<0.001		
Malignant neoplasm of breast (n = 1099)			
No. of cases/person-years	196/179151	685/516912	218/186909
HR (95% CI)	1.00 (ref)	1.01(086-1.19)	0.88(0.72-1.07)
p-value		0.874	0.195
p-value for trend	0.184		
Malignant neoplasm of other and ill-defined sites (n = 7363)			
No. of cases/person-years	1408/179151	4357/516912	1598/186909
HR (95% CI)	1.00 (ref)	0.99(0.93-1.05)	0.97(0.90-1.04)
p-value		0.795	0.405
p-value for trend	0.663		

[Cox proportional hazards regression adjusted for age at baseline, sex, ethnicity, education, employment status, TDI, BMI categories; medication treatment history for anti-cholesterol drug, antihypertensive drug, and insulin; family history of hypertension, DM, and cancer; and the number of MetS component traits. Unfavorable lifestyle (lowest quintile of weighted healthy lifestyle scores) was considered the reference group.]

Table S8. HR (95% CI) for all-cause and cause-specific mortality by unweighted healthy lifestyle scores

	Unweighted lifestyle scores					
Endpoints	0-1 (n = 2243)	2 (n = 7875)	3 (n = 17408)	4 (n = 24083)	5 (n = 21609)	6-7 (n = 14124)
All-cause mortality (n = 6739)						
No. of cases/person-years	325/27290	842/96507	1596/213589	1854/296327	1372/266304	750/174611
HR (95% CI)	1.00 (ref)	0.72(0.63-0.82)	0.64(0.57-0.72)	0.57(0.50-0.64)	0.49(0.44-0.56)	0.45(0.39-0.51)
p-value		<0.001	<0.001	<0.001	<0.001	<0.001
p-value for trend	<0.001					
Cancer (n = 2712)						
No. of cases/person-years	121/27290	326/96507	645/213589	710/296327	570/266304	340/174611
HR (95% CI)	1.00 (ref)	0.74(0.60-0.91)	0.67(0.55-0.82)	0.55(0.45-0.67)	0.51(0.42-0.62)	0.49(0.40-0.61)
p-value		0.004	<0.001	<0.001	<0.001	<0.001
p-value for trend	<0.001					
Cardiovascular Disease (n = 1731)						
No. of cases/person-years	81/27290	219/96507	430/213589	474/296327	362/266304	165/174611
HR (95% CI)	1.00 (ref)	0.76(0.59-0.98)	0.72(0.57-0.92)	0.62(0.49-0.79)	0.57(0.45-0.73)	0.45(0.35-0.60)
p-value		0.034	0.007	<0.001	<0.001	<0.001
p-value for trend	<0.001					
Respiratory disease (n = 535)						
No. of cases/person-years	40/27290	72/96507	127/213589	171/296327	82/266304	43/174611
HR (95% CI)	1.00 (ref)	0.52(0.36-0.77)	0.45(0.32-0.65)	0.48(0.34-0.68)	0.28(0.19-0.41)	0.25(0.16-0.39)
p-value		0.004	<0.001	<0.001	<0.001	<0.001
p-value for trend	<0.001					
Digestive disease (n = 321)						

No. of cases/person-years	24/27290	53/96507	76/213589	91/296327	54/266304	23/174611
HR (95% CI)	1.00 (ref)	0.63(0.39-1.02)	0.43(0.27-0.69)	0.40(0.25-0.63)	0.28(0.17-0.46)	0.20(0.11-0.36)
p-value		0.063	<0.001	<0.001	<0.001	<0.001
p-value for trend	<0.001					
Other causes (n = 1440)						
No. of cases/person-years	59/27290	172/96507	318/213589	408/296327	304/266304	179/174611
HR (95% CI)	1.00 (ref)	0.80(0.59-1.07)	0.69(0.52-0.91)	0.67(0.51-0.88)	0.58(0.44-0.77)	0.56(0.41-0.75)
p-value		0.136	0.009	0.004	<0.001	<0.001
p-value for trend	<0.001					

[Cox proportional hazards regression adjusted for age at baseline, sex, ethnicity, education, employment status, TDI, BMI categories; medication treatment history for anti-cholesterol drug, antihypertensive drug, and insulin; family history of hypertension, DM, and cancer; and the number of MeTs component traits. 0-1 lifestyle score was considered the reference group.]

Table S9. HR (95% CI) for all-cause and cause-specific mortality by weighted lifestyle categories

Endpoints	Lifestyle category (weighted lifestyle scores for mortality)		
	Unfavorable (Quintile 1; n = 17697)	Intermedium (Quintile 2-4; n = 48699)	Favorable (Quintile 5; n = 20946)
**All-cause mortality** (n = 6739)			
No. of cases/person-years	1961/216483	3587/599653	1191/258494
HR (95% CI)	1.00 (ref)	0.66(0.63-0.70)	0.57(0.53-0.62)
p-value		<0.001	<0.001
p-value for trend	<0.001		
**Cancer** (n = 2712)			
No. of cases/person-years	781/216483	1408/599653	523/258494
HR (95% CI)	1.00 (ref)	0.63(0.58-0.69)	0.59(0.53-0.66)
p-value		<0.001	<0.001
p-value for trend	<0.001		
**Cardiovascular disease** (n = 1731)			
No. of cases/person-years	521/216483	932/599653	278/258494
HR (95% CI)	1.00 (ref)	0.67(0.60-0.75)	0.54(0.47-0.63)
p-value		<0.001	<0.001
p-value for trend	<0.001		
**Respiratory disease** (n = 535)			
No. of cases/person-years	178/216483	287/599653	70/258494
HR (95% CI)	1.00 (ref)	0.62(0.52-0.76)	0.42(0.31-0.55)
p-value		<0.001	<0.001
p-value for trend	<0.001		
**Digestive disease** (n = 321)			
No. of cases/person-years	115/216483	168/599653	38/258494
HR (95% CI)	1.00 (ref)	0.55(0.43-0.70)	0.33(0.23-0.48)
p-value		<0.001	<0.001
p-value for trend	<0.001		
**Other causes** (n = 1440)			
No. of cases/person-years	366/216483	792/599653	282/258494
HR (95% CI)	1.00 (ref)	0.78(0.69-0.88)	0.72(0.61-0.84)
p-value		<0.001	<0.001
p-value for trend	<0.001		

[Cox proportional hazards regression adjusted for age at baseline, sex, ethnicity, education, employment status, TDI, BMI categories; medication treatment history for anti-cholesterol drug, antihypertensive drug, and insulin; family history of hypertension, DM, and cancer; and the number of MetS component traits. Unfavorable lifestyle (lowest quintile of weighted lifestyle scores) was considered the reference group.]

Table S10. Crossover analysis of interaction between lifestyle categories (weighted lifestyle scores) and baseline characteristics on all-cause mortality risk

Subgroup	No. of death cases	HR (95% CI)	*p*-value	*p*-trend
**Age at baseline**				<0.001
Age 40-49 years				
Unfavorable	3811(4.4)	1.00 (ref)		
Intermediate	8356(9.6)	0.53(0.42-0.67)	<0.001	
Favorable	4097(4.7)	0.53(0.40-0.72)	<0.001	
Age 50-59 years				
Unfavorable	6240(7.1)	0.71(0.58-0.87)	0.001	
Intermediate	16134(18.5)	0.46(0.37-0.56)	<0.001	
Favorable	6646(7.6)	0.39(0.31-0.49)	<0.001	
Age ≥60 years				
Unfavorable	7646(8.8)	0.68(0.54-0.87)	0.002	
Intermediate	24209(27.7)	0.47(0.37-0.59)	<0.001	
Favorable	10203(11.7)	0.40(0.31-0.51)	<0.001	
**Sex**				<0.001
Male				
Unfavorable	11096(12.7)	1.00 (ref)		
Intermediate	26546(30.4)	0.72(0.68-0.77)	<0.001	
Favorable	10192(11.7)	0.63(0.58-0.70)	<0.001	
Female				
Unfavorable	6601(7.6)	0.80(0.72-0.88)	<0.001	
Intermediate	22153(25.4)	0.43(0.40-0.47)	<0.001	
Favorable	10754(12.3)	0.37(0.33-0.41)	0.003	
**Employment status**				<0.001
Unemployed				
Unfavorable	2536(2.9)	1.00 (ref)		
Intermediate	4230(4.8)	0.56(0.49-0.65)	<0.001	
Favorable	1436(1.6)	0.50(0.39-0.63)	<0.001	
Employed				
Unfavorable	15161(17.4)	0.54(0.48-0.61)	<0.001	
Intermediate	44469(50.9)	0.37(0.33-0.42)	<0.001	
Favorable	19510(22.3)	0.32(0.28-0.36)	<0.001	
**TDI quintile**				<0.001
5(most deprived)				
Unfavorable	4866(5.6)	1.00 (ref)		
Intermediate	9146(10.5)	0.67(0.61-0.74)	<0.001	
Favorable	3455(4.0)	0.49(0.42-0.57)	<0.001	
2- 4 quintile				
Unfavorable	9986(11.4)	0.69(0.63-0.76)	<0.001	
Intermediate	29530(33.8)	0.46(0.43-0.51)	<0.001	
Favorable	12879(14.7)	0.42(0.38-0.47)	<0.001	
1(least deprived)				
Unfavorable	2845(3.3)	0.66(0.58-0.76)	<0.001	
Intermediate	10023(11.5)	0.42(0.38-0.47)	<0.001	
Favorable	4612(5.3)	0.37(0.32-0.43)	<0.001	
**BMI categories**				<0.001

<25 kg/m^2				
Unfavorable	1179(1.3)	1.00 (ref)		
Intermediate	2987(3.4)	0.42(0.34-0.52)	<0.001	
Favorable	1973(2.3)	0.29(0.22-0.38)	<0.001	
<30 kg/m^2				
Unfavorable	6778(7.8)	0.72(0.61-0.85)	<0.001	
Intermediate	18504(21.2)	0.45(0.38-0.53)	<0.001	
Favorable	8962(10.3)	0.40(0.33-0.48)	<0.001	
≥30 kg/m^2				
Unfavorable	9740(11.2)	0.70(0.60-0.83)	<0.001	
Intermediate	27208(31.2)	0.51(0.43-0.60)	<0.001	
Favorable	10011(11.5)	0.45(0.38-0.54)	<0.001	
**Family history of hypertension**				<0.001
No				
Unfavorable	9325(10.7)	1.00 (ref)		
Intermediate	24061(27.5)	0.68(0.63-0.73)	<0.001	
Favorable	10218(11.7)	0.57(0.52-0.63)	<0.001	
Yes				
Unfavorable	8372(9.6)	0.93(0.85-1.02)	0.143	
Intermediate	24638(28.2)	0.60(0.56-0.65)	<0.001	
Favorable	10728(12.3)	0.54(0.48-0.59)	<0.001	
**Family history of DM**				<0.001
No				
Unfavorable	12959(14.8)	1.00 (ref)		
Intermediate	35211(40.3)	0.64(0.60-0.69)	<0.001	
Favorable	15040(17.2)	0.56(0.52-0.61)	<0.001	
Yes				
Unfavorable	4738(5.4)	0.90(0.81-1.00)	0.053	
Intermediate	13488(15.4)	0.66(0.61-0.71)	<0.001	
Favorable	5906(6.8)	0.55(0.49-0.63)	<0.001	
**Family history of Cancer**				<0.001
No				
Unfavorable	11469(13.1)	1.00 (ref)		
Intermediate	31286(35.8)	0.66(0.61-0.71)	<0.001	
Favorable	13951(16.0)	0.58(0.53-0.64)	<0.001	
Yes				
Unfavorable	6228(7.1)	0.98(0.90-1.08)	0.721	
Intermediate	17413(19.9)	0.66(0.61-0.71)	<0.001	
Favorable	6995(8.0)	0.55(0.49-0.62)	<0.001	

[Abbreviation: TDI, Townsend deprivation index; FH, family history; DM, diabetes mellitus; Unfavorable, lowest quintile of weighted healthy lifestyle scores; Intermediate, quintiles 2-4 of weighted healthy lifestyle scores; Favorable, highest quintile of weighted healthy lifestyle scores. Cox proportional hazards regression adjusted for age at baseline, sex and ethnicity, education, employment status and TDI, BMI; medication treatment history for anti-cholesterol drug, antihypertensive drug, and insulin; family history of hypertension, DM, and cancer; and the number of MeTs component traits (excluded the corresponding variable when stratified by itself).]

Table S11. Association of unweighted lifestyle scores category with all-cause and cause-specific mortality risk

Endpoints	Lifestyle category (unweighted lifestyle scores)		
	Scoring 0-2 (n = 10118)	Scoring 3-5 (n = 63100)	Scoring 6-7 (n = 14124)
**All-cause mortality** (n = 6739)			
No. of cases/person-years	1167/123797	4822/776221	750/174611
HR (95% CI)	1.00 (ref)	0.73(0.68-0.78)	0.58(0.53-0.64)
p-value		<0.001	<0.001
p-value for trend	<0.001		
**Cancer** (n = 2712)			
No. of cases/person-years	447/123797	1925/776221	340/174611
HR (95% CI)	1.00 (ref)	0.72(0.65-0.80)	0.63(0.54-0.72)
p-value		<0.001	<0.001
p-value for trend	<0.001		
**Cardiovascular disease** (n = 1731)			
No. of cases/person-years	300/123797	1266/776221	165/174611
HR (95% CI)	1.00 (ref)	0.79(0.69-0.90)	0.57(0.47-0.69)
p-value		<0.001	<0.001
p-value for trend	<0.001		
**Respiratory disease** (n = 535)			
No. of cases/person-years	112/123797	380/776221	43/174611
HR (95% CI)	1.00 (ref)	0.65(0.53-0.81)	0.40(0.28-0.58)
p-value		<0.001	<0.001
p-value for trend	<0.001		
**Digestive disease** (n = 321)			
No. of cases/person-years	77/123797	221/776221	23/174611
HR (95% CI)	1.00 (ref)	0.52(0.40-0.68)	0.29(0.18-0.46)
p-value		<0.001	<0.001
p-value for trend	<0.001		
**Other causes** (n = 1440)			
No. of cases/person-years	231/123797	1030/776221	179/174611
HR (95% CI)	1.00 (ref)	0.77(0.67-0.89)	0.67(0.55-0.82)
p-value		<0.001	<0.001
p-value for trend	<0.001		

[Cox proportional hazards regression adjusted for age at baseline, sex, ethnicity, education, employment status, TDI, BMI categories; medication treatment history for anti-cholesterol drug, antihypertensive drug, and insulin; family history of hypertension, DM, and cancer; and the number of MetS component

traits. 0-1 lifestyle score was considered the reference group.]

Table S12. Association of unweighted lifestyle scores category with overall and site-specific cancer risk

Endpoints	Lifestyle category (unweighted lifestyle scores)		
	Scoring 0-2 (n = 10118)	Scoring 3-5 (n = 63100)	Scoring 6-7 (n = 14124)
Overall cancer (n = 10802)			
No. of cases/person-years	1370/101669	7784/638033	1648/143271
HR (95% CI)	1.00 (ref)	0.93(0.87-0.98)	0.93(0.87-1.01)
p-value		0.010	0.070
p-value for trend	0.035		
Malignant neoplasms of digestive organs (n = 1665)			
No. of cases/person-years	242/101669	1212/638033	211/143271
HR (95% CI)	1.00 (ref)	0.86(0.75-0.99)	0.78(0.64-0.94)
p-value		0.039	0.009
p-value for trend	0.028		
Malignant neoplasms of respiratory and intrathoracic organs (n = 675)			
No. of cases/person-years	158/101669	457/638033	60/143271
HR (95% CI)	1.00 (ref)	0.49(0.41-0.59)	0.31(0.23-0.42)
p-value		<0.001	<0.001
p-value for trend	<0.001		
Malignant neoplasm of breast (n = 1099)			
No. of cases/person-years	115/101669	758/638033	226/143271
HR (95% CI)	1.00 (ref)	0.78(0.64-0.95)	0.80(0.64-1.01)
p-value		0.014	0.060
p-value for trend	0.048		

[Cox proportional hazards regression adjusted for age at baseline, sex, ethnicity, education, employment status, TDI, BMI categories; medication treatment history for anti-cholesterol drug, antihypertensive drug, and insulin; family history of hypertension, DM, and cancer; and the number of MetS component traits. 0-1 lifestyle score was considered the reference group.].

Table S13. HR (95% CI) of all-cause and cause-specific mortality risk according to unweighted lifestyle category with further inclusion of BMI

Endpoints	Lifestyle category (unweighted lifestyle scores)		
	Scoring 0-2; (n = 9758)	Scoring 3-5; (n = 61763)	Scoring 6-8; (n = 15821)
**All-cause mortality** (n = 6739)			
No. of cases/person-	1125/119437	4783/759686	831/195507
HR (95% CI)	1.00 (ref)	0.73(0.69-0.78)	0.56(0.51-0.62)
p-value		<0.001	<0.001
p-value for trend	<0.001		
**Cancer** (n = 2712)			
No. of cases/person-	431/119437	1911/759686	370/195507
HR (95% CI)	1.00 (ref)	0.73(0.66-0.81)	0.60(0.52-0.70)
p-value		<0.001	<0.001
p-value for trend	<0.001		
**Cardiovascular disease** (n = 1731)			
No. of cases/person-	289/119437	1258/759686	184/195507
HR (95% CI)	1.00 (ref)	0.78(0.68-0.89)	0.55(0.45-0.67)
p-value		<0.001	<0.001
p-value for trend	<0.001		
**Respiratory disease** (n = 535)			
No. of cases/person-	105/119437	379/759686	51/195507
HR (95% CI)	1.00 (ref)	0.67(0.54-0.84)	0.39(0.27-0.56)
p-value		<0.001	<0.001
p-value for trend	<0.001		
**Digestive disease** (n = 321)			
No. of cases/person-	74/119437	221/759686	26/195507
HR (95% CI)	1.00 (ref)	0.53(0.41-0.69)	0.27(0.17-0.44)
p-value		<0.001	<0.001
p-value for trend	<0.001		
**Other causes** (n = 1440)			
No. of cases/person-	226/119437	1014/759686	200/195507
HR (95% CI)	1.00 (ref)	0.76(0.65-0.88)	0.65(0.53-0.79)
p-value		<0.001	<0.001
p-value for trend	<0.001		

[Cox proportional hazards regression adjusted for age at baseline, sex, ethnicity, education, employment status, TDI, medication treatment history for anti-cholesterol drug, antihypertensive drug, and insulin; family history of hypertension, DM, and cancer; and the number of MetS component traits. 0-2 lifestyle score was considered the reference group. 18.5 ≤ BMI <25 defined as healthy level].

Table S14. HR (95% CI) of overall and site-specific cancer risk according to unweighted lifestyle category with further inclusion of BMI

Endpoints	Lifestyle category (unweighted lifestyle scores)		
	Scoring 0-2; (n = 9758)	Scoring 3-5; (n = 61763)	Scoring 6-8; (n = 15821)
**Overall cancer** (n = 10802)			
No. of cases/person-years	1325/98063	7646/624416	1831/160493
HR (95% CI)	1.00 (ref)	0.93(0.87-0.98)	0.92(0.86-0.99)
p-value		0.011	0.035
p-value for trend	0.035		
**Malignant neoplasms of digestive organs** (n = 1665)			
No. of cases/person-years	234/98063	1205/624416	226/160493
HR (95% CI)	1.00 (ref)	0.88(0.76-1.01)	0.75(0.62-0.91)
p-value		0.064	0.003
p-value for trend	0.014		
**Malignant neoplasms of respiratory and intrathoracic organs** (n = 675)			
No. of cases/person-years	153/98063	454/624416	68/160493
HR (95% CI)	1.00 (ref)	0.48(0.40-0.58)	0.28(0.21-0.38)
p-value		<0.001	<0.001
p-value for trend	<0.001		
**Malignant neoplasm of breast** (n = 1099)			
No. of cases/person-years	110/98063	740/624416	249/160493
HR (95% CI)	1.00 (ref)	0.78(0.64-0.96)	0.81(0.64-1.01)
p-value		0.018	0.064
p-value for trend	0.060		

[Cox proportional hazards regression adjusted for age at baseline, sex, ethnicity, education, employment status, TDI, medication treatment for cholesterol, hypertension, and DM; medication treatment history for anti-cholesterol drug, antihypertensive drug, and insulin; and the number of MetS component traits. 0-2 lifestyle score was considered the reference group. 18.5 ≤ BMI <25 defined as healthy level].

Table S15. HR (95% CI) of all-cause and cause-specific mortality risk according to unweighted lifestyle category with further redefining never smoke as a healthy behavior

Endpoints	Lifestyle category (unweighted lifestyle scores)		
	Scoring 0-2; (n = 17455)	Scoring 3-5; (n = 59809)	Scoring 6-7; (n = 10078)
**All-cause mortality** (n = 6739)			
No. of cases/person-	1970/213287	4326/736497	443/124846
HR (95% CI)	1.00 (ref)	0.76(0.72-0.80)	0.55(0.49-0.61)
p-value		<0.001	<0.001
p-value for trend	<0.001		
**Cancer** (n = 2712)			
No. of cases/person-	747/213287	1762/736497	203/124846
HR (95% CI)	1.00 (ref)	0.78(0.71-0.85)	0.61(0.52-0.71)
p-value		<0.001	<0.001
p-value for trend	<0.001		
**Cardiovascular disease** (n = 1731)			
No. of cases/person-	512/213287	1115/736497	104/124846
HR (95% CI)	1.00 (ref)	0.81(0.73-0.90)	0.58(0.46-0.71)
p-value		<0.001	<0.001
p-value for trend	<0.001		
**Respiratory disease** (n = 535)			
No. of cases/person-years	189/213287	330/736497	16/124846
HR (95% CI)	1.00 (ref)	0.66(0.55-0.80)	0.24(0.14-0.40)
p-value		<0.001	<0.001
p-value for trend	<0.001		
**Digestive disease** (n = 321)			
No. of cases/person-	118/213287	190/736497	13/124846
HR (95% CI)	1.00 (ref)	0.57(0.45-0.72)	0.28(0.16-0.50)
p-value		<0.001	<0.001
p-value for trend	<0.001		
**Other causes** (n = 1440)			
No. of cases/person-	404/213287	929/736497	107/124846
HR (95% CI)	1.00 (ref)	0.77(0.69-0.87)	0.60(0.49-0.75)
p-value		<0.001	<0.001
p-value for trend	<0.001		

[Cox proportional hazards regression adjusted for age at baseline, sex, ethnicity, education, employment status, TDI, BMI categories; medication treatment history for anti-cholesterol drug, antihypertensive drug, and insulin; family history of hypertension, DM, and cancer; and the number of MetS component traits. 0-1 lifestyle score was considered the reference group.]

Table S16. HR (95% CI) of overall and site-specific cancer risk according to unweighted lifestyle category with further redefining never smoke as a healthy behavior

	Lifestyle category (unweighted lifestyle scores)		
Endpoints	Scoring 0-2; (n = 17455)	Scoring 3-5; (n = 59809)	Scoring 6-7; (n = 10078)
Overall cancer (n = 10802)			
No. of cases/person-years	2433/175212	7285/605122	1084/102639
HR (95% CI)	1.00 (ref)	0.95(0.91-0.99)	0.93(0.86-0.99)
p-value		0.039	0.038
p-value for trend	0.057		
Malignant neoplasms of digestive organs (n = 1665)			
No. of cases/person-years	424/175212	1117/605122	124/102639
HR (95% CI)	1.00 (ref)	0.89(0.80-1.00)	0.71(0.57-0.87)
p-value		0.054	0.001
p-value for trend	0.003		
Malignant neoplasms of respiratory and intrathoracic organs (n = 675)			
No. of cases/person-years	238/175212	410/605122	27/102639
HR (95% CI)	1.00 (ref)	0.56(0.48-0.67)	0.24(0.16-0.36)
p-value		<0.001	<0.001
p-value for trend	<0.001		
Malignant neoplasm of breast (n = 1099)			
No. of cases/person-years	181/175212	756/605122	162/102639
HR (95% CI)	1.00 (ref)	0.88(0.75-1.04)	0.86(0.69-1.07)
p-value		0.136	0.176
p-value for trend	0.284		

[Cox proportional hazards regression adjusted for age at baseline, sex, ethnicity, education, employment status, TDI, BMI categories; medication treatment history for anti-cholesterol drug, antihypertensive drug, and insulin; family history of hypertension, DM, and cancer; and the number of MetS component traits. 0-1 lifestyle score was considered the reference group.]

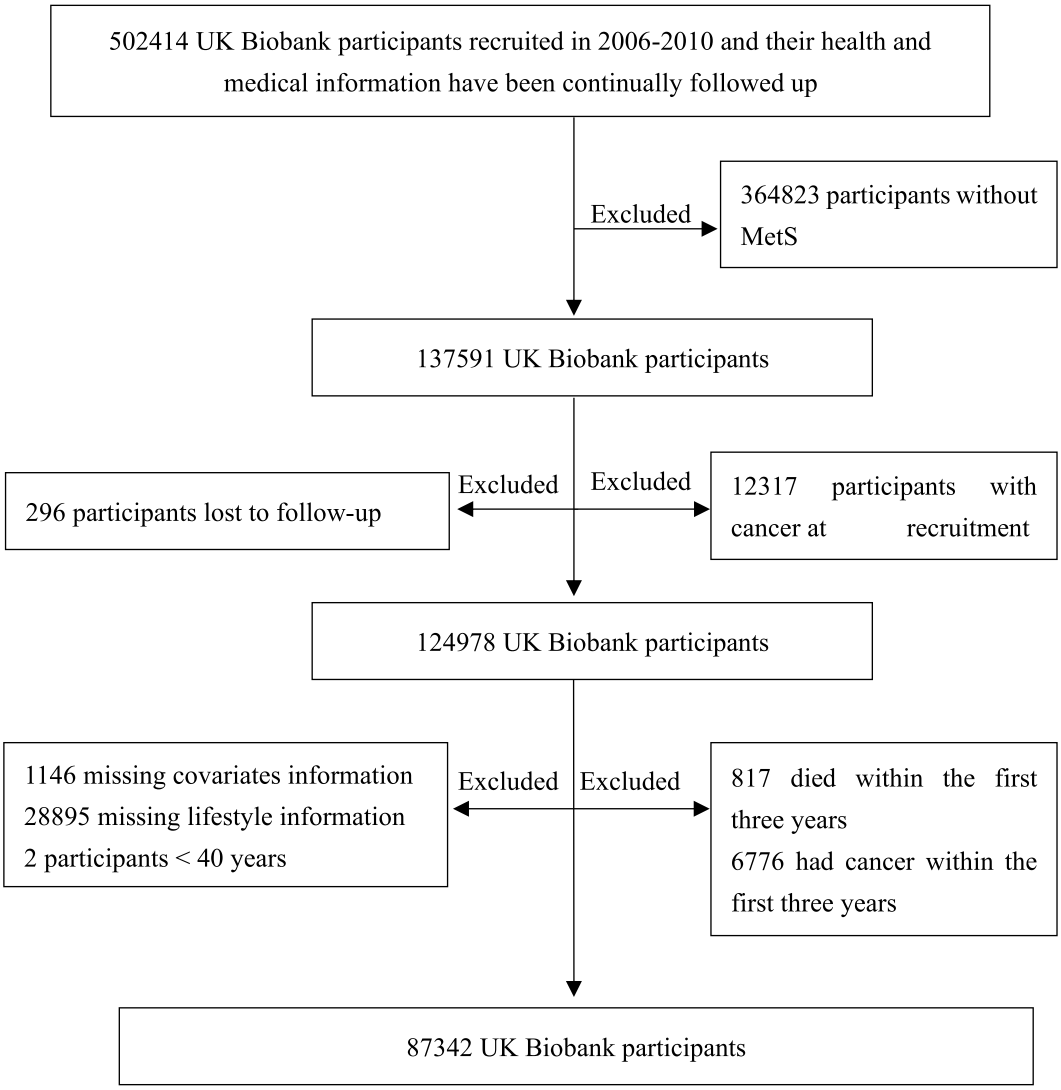

Figure. S1 Cohort exclusions of the study participants

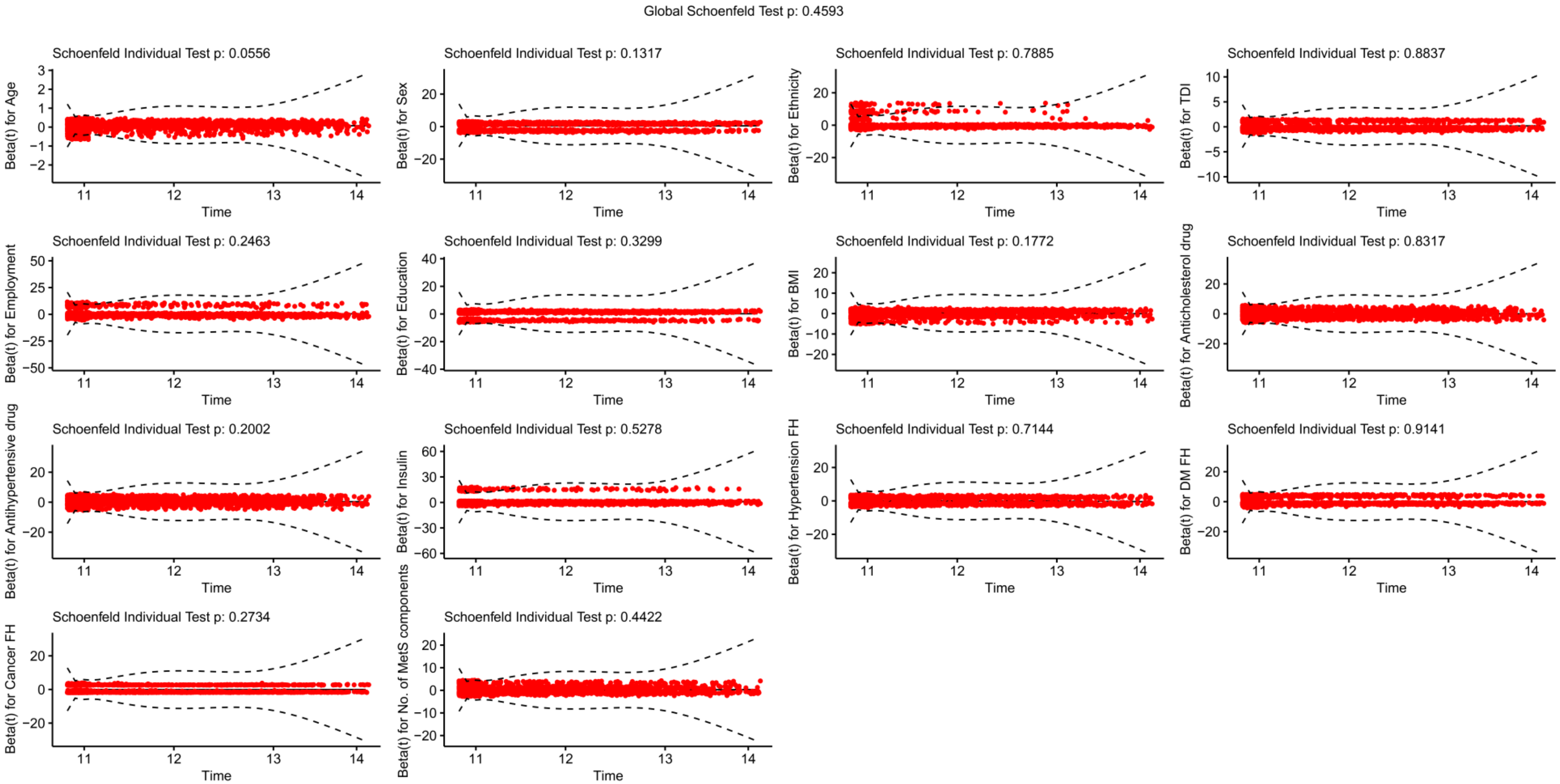

Figure. S2 Schoenfeld residuals test for all-cause mortality

Abbreviations: TDI, Townsend deprivation index; BMI, body mass index; FH, family history; DM, diabetes mellitus; MetS, metabolic syndrome.

The unit of time is years.

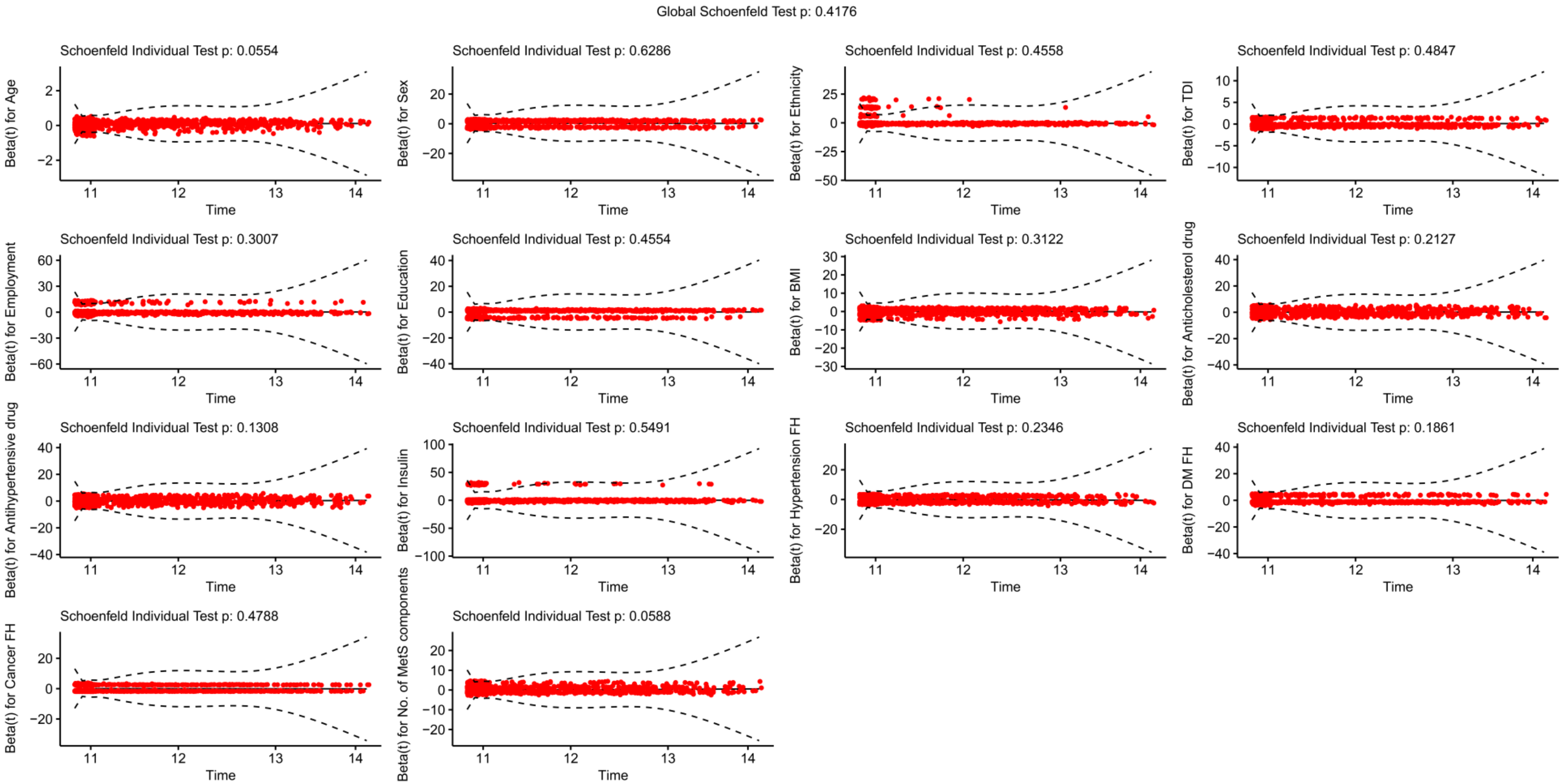

Figure. S3 Schoenfeld residuals test for cancer mortality

Abbreviations: TDI, Townsend deprivation index; BMI, body mass index; FH, family history; DM, diabetes mellitus; MetS, metabolic syndrome.

The unit of time is years.

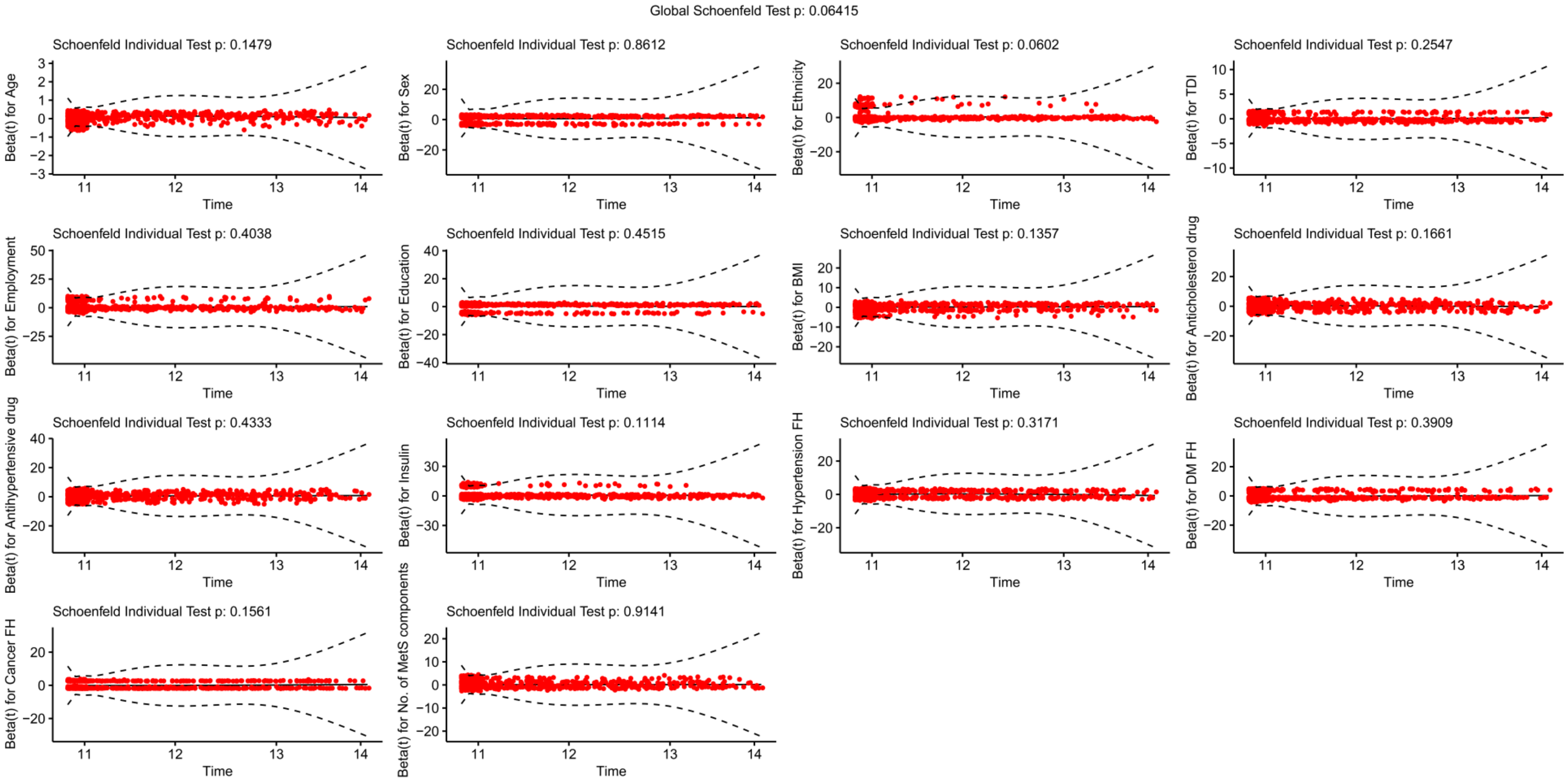

Figure. S4 Schoenfeld residuals test for cardiovascular disease mortality

Abbreviations: TDI, Townsend deprivation index; BMI, body mass index; FH, family history; DM, diabetes mellitus; MetS, metabolic syndrome.

The unit of time is years.

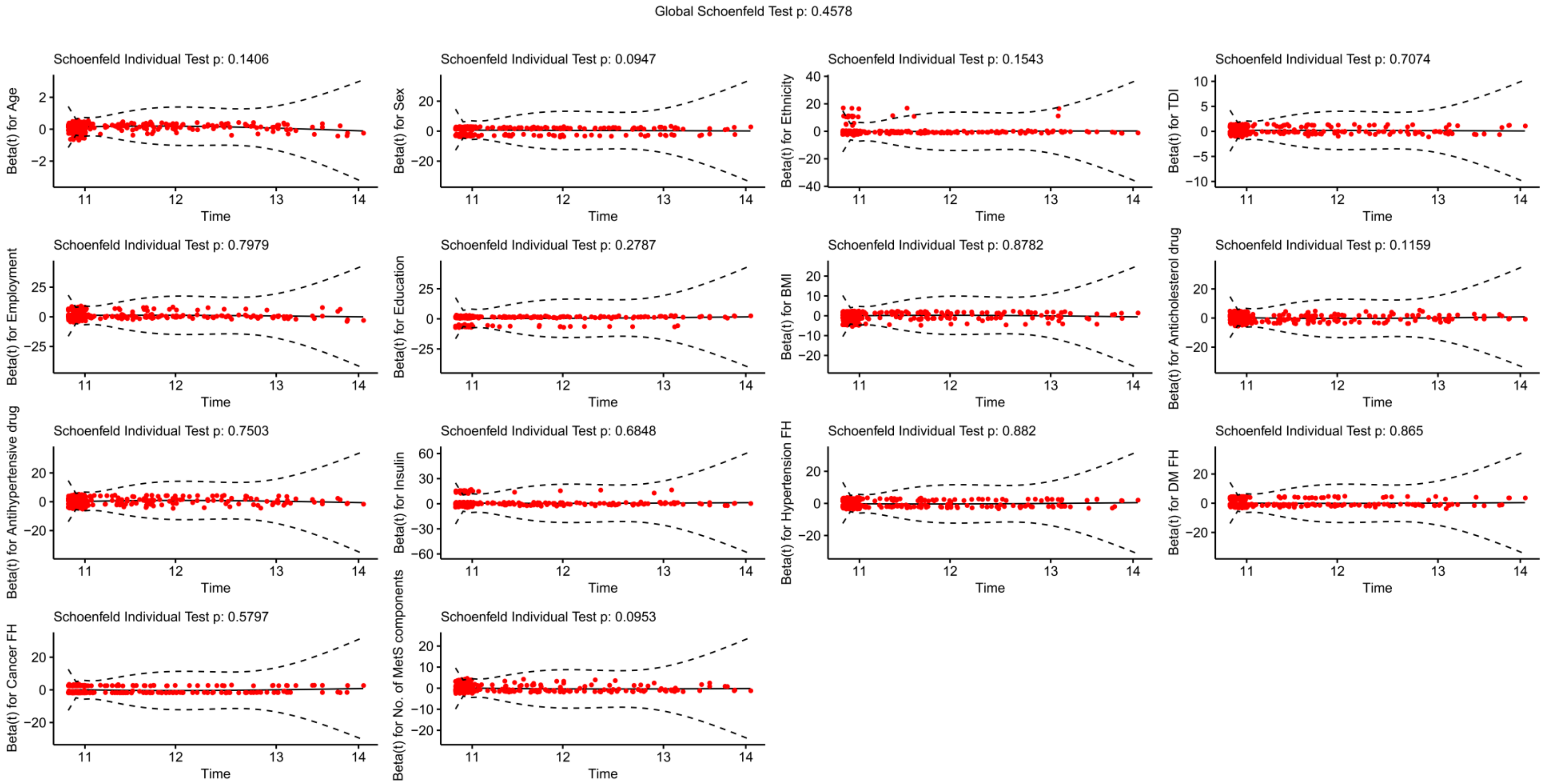

Figure. S5 Schoenfeld residuals test for respiratory disease mortality

Abbreviations: TDI, Townsend deprivation index; BMI, body mass index; FH, family history; DM, diabetes mellitus; MetS, metabolic syndrome.

The unit of time is years.

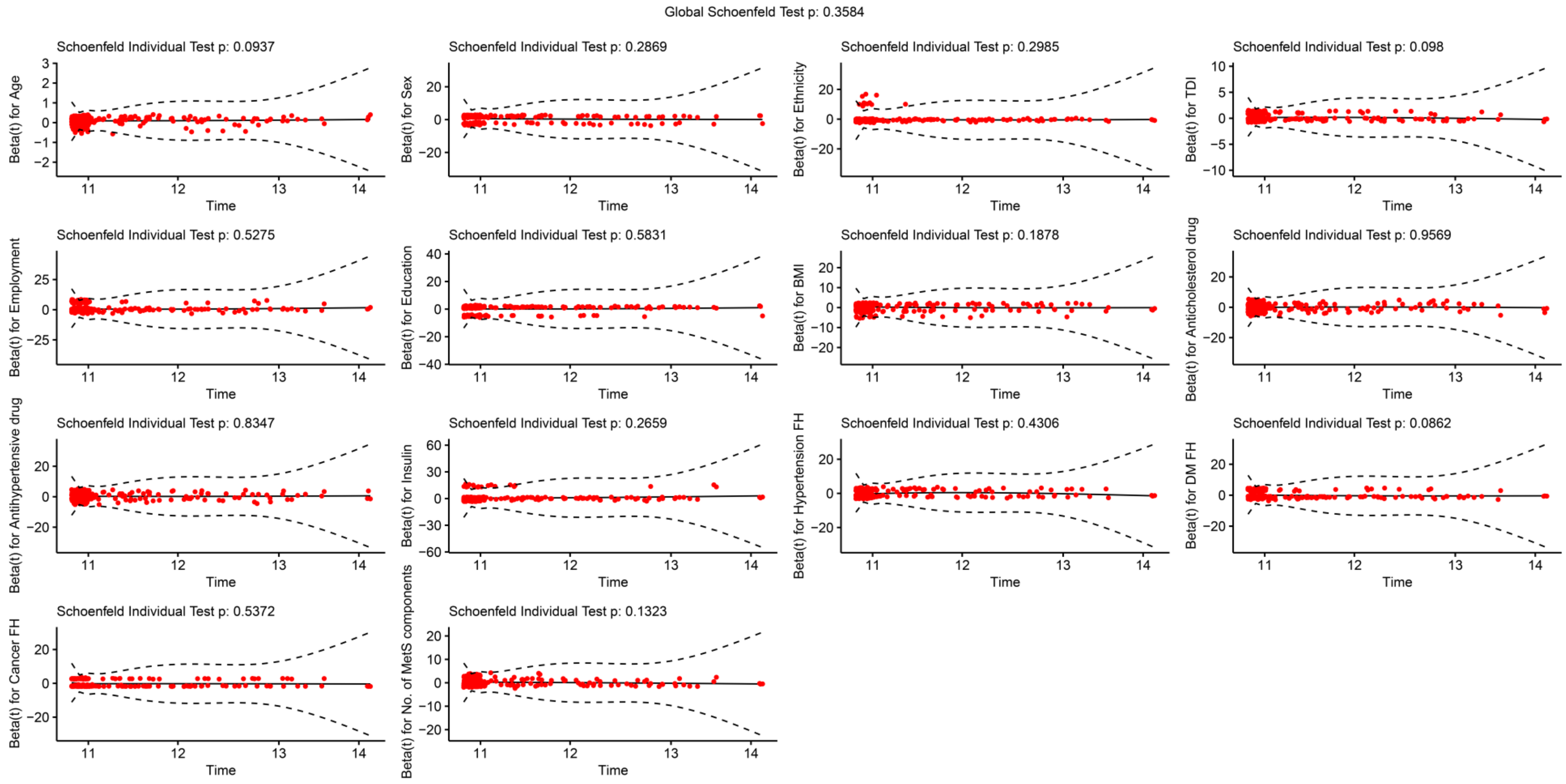

Figure. S6 Schoenfeld residuals test for digestive disease mortality

Abbreviations: TDI, Townsend deprivation index; BMI, body mass index; FH, family history; DM, diabetes mellitus; MetS, metabolic syndrome.

The unit of time is years.

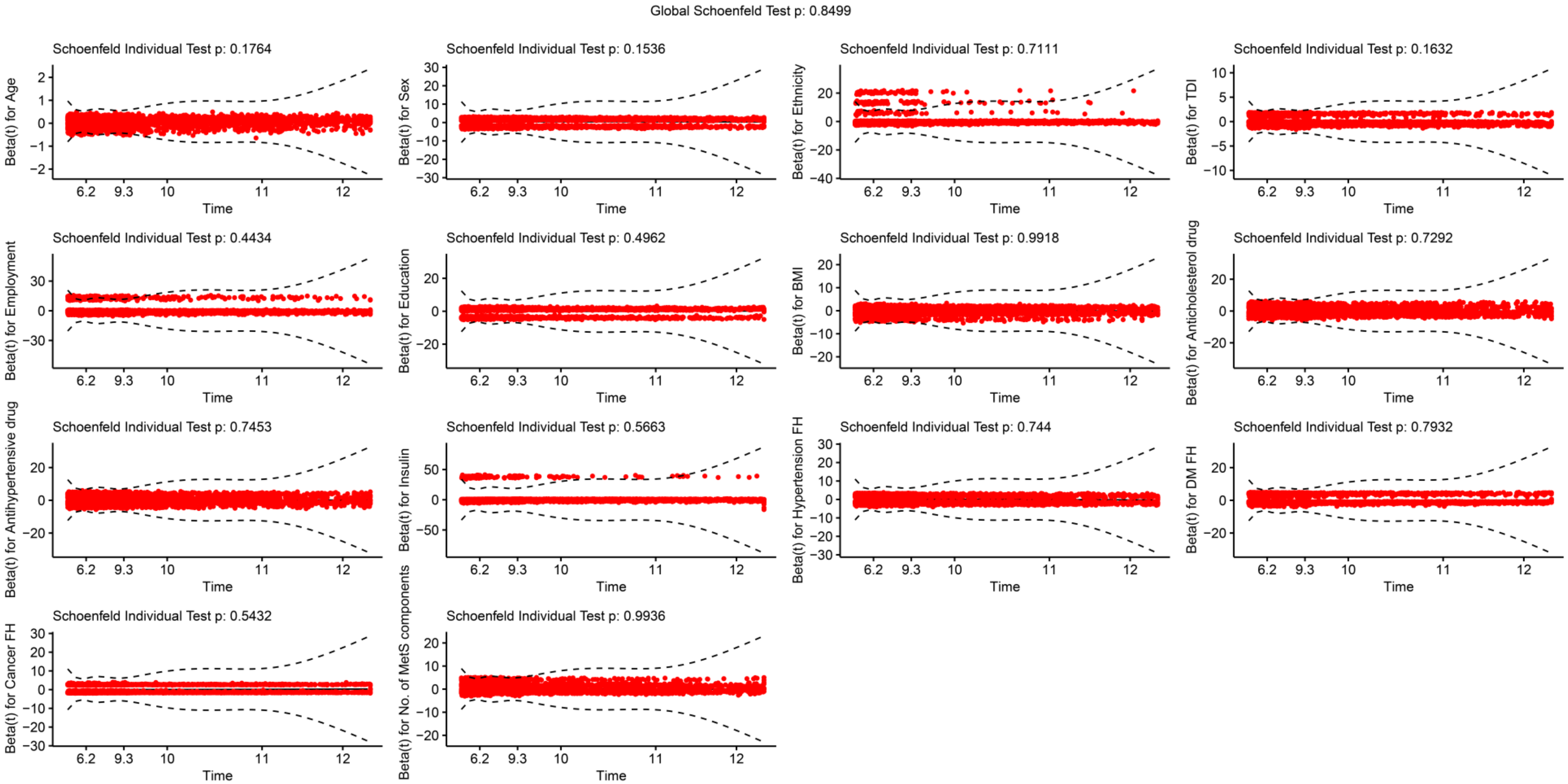

Figure. S7 Schoenfeld residuals test for overall incident cancer

Abbreviations: TDI, Townsend deprivation index; BMI, body mass index; FH, family history; DM, diabetes mellitus; MetS, metabolic syndrome.

The unit of time is years.

(a) Unweighted healthy lifestyle scores

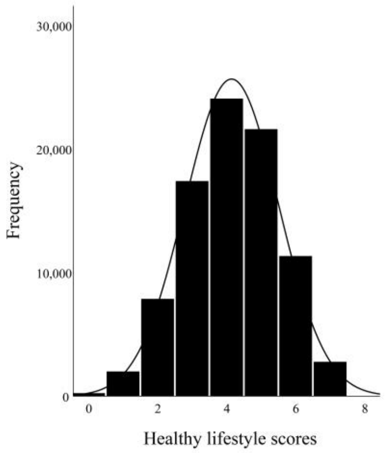

(b) Healthy lifestyle scores weighted by all-cause mortality outcome

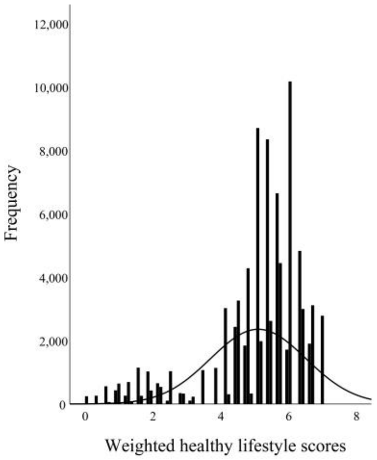

(c) Healthy lifestyle scores weighted by overall cancer incidence outcome

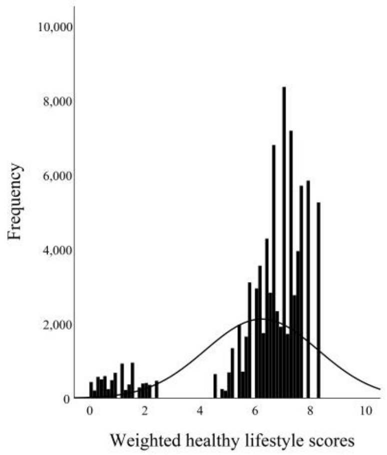

(d) Unweighted healthy lifestyle scores with further inclusion of BMI

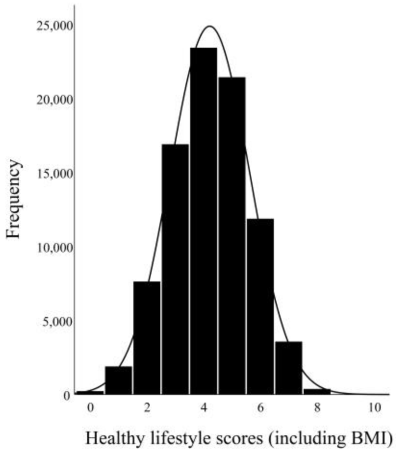

(e) Unweighted healthy lifestyle scores with further redefining never smoke as a healthy behavior

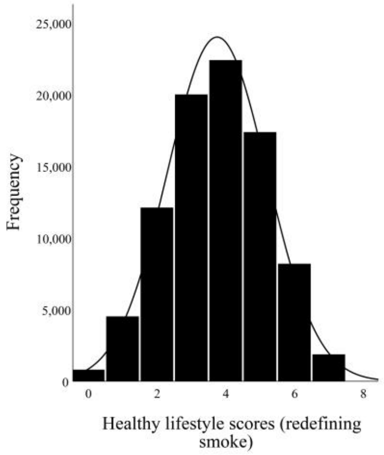

[Abbreviation: BMI, body mass index. (a) Unweighted healthy lifestyle scores, including *never/quitting smoking, moderate drinking, good sleep, healthy diet, sufficient exercise, social support*, and *less sedentary behaviour*; (b) Weighted healthy lifestyle scores by considering each β lifestyle factor coefficient on all-cause mortality effect; (c) Weighted healthy lifestyle scores by considering each β lifestyle factor coefficient on overall cancer effect; (d) Unweighted healthy lifestyle scores with further inclusion of 18.5 ≤ BMI <25 as healthy level; (e) Unweighted healthy lifestyle scores with further redefining never smoke as a healthy behavior.]

Figure. S8 Distribution of the healthy lifestyle scores

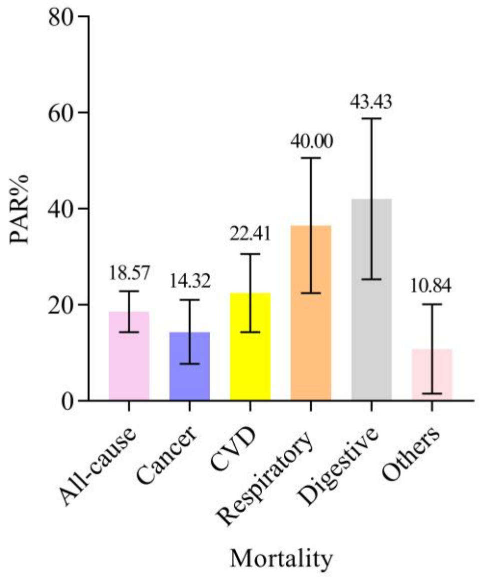

Figure. S9 Multivariable-adjusted population-attributable risk percents (95% CI) for all-cause and cause-specific mortality according to weighted lifestyle scores

[The PAR% of all-cause and cause-specific mortality attributable to nonadherence to a favorable lifestyle ((highest quintile of weighted lifestyle scores). The multivariable model was adjusted for age at baseline, sex, ethnicity, education, employment status, TDI, BMI categories; medication treatment history for anti-cholesterol drug, antihypertensive drug, and insulin; family history of hypertension, DM, and cancer; and the number of MetS component traits. Abbreviations: PAR%, population-attributable risk percents; CI, confidence interval; CVD, cardiovascular disease.]
